# HTLV-1 biofilm polarization maintained by tetraspanin CD82 is required for efficient viral transmission

**DOI:** 10.1128/mbio.01326-23

**Published:** 2023-10-27

**Authors:** Coline Arone, Samuel Martial, Julien Burlaud-Gaillard, Maria-Isabel Thoulouze, Philippe Roingeard, Hélène Dutartre, Delphine Muriaux

**Affiliations:** 1 Infectious Disease Research Institute of Montpellier (IRIM), UMR CNRS, Montpellier, France; 2 Center for International Research on Infectiology (CIRI), UMR Inserm, Lyon, France; 3 IBiSA Electron Microscopy Platform of Tours University, UMR Inserm, Tours, France; 4 Plateforme d'Infectiologie Expérimentale (PFIE), UE INRAE, Nouzilly, France; University of North Carolina, Chapel Hill, North Carolina, USA

**Keywords:** HTLV-1, Gag, viral biofilms, cell-to-cell viral transmission, tetraspanins, CD82, N-glycosylation, NGI-1, super-resolution microscopy

## Abstract

The human T-lymphotropic virus type 1 (HTLV-1) is an oncogenic retrovirus whose transmission relies primarily on cell-to-cell contacts as cell-free viruses are poorly infectious. Among the intercellular transmission routes described, HTLV-1 biofilms are adhesive structures polarized at the cell surface that confine virions in a protective environment, which is believed to promote their simultaneous delivery during infection. Here, we show that several tetraspanins are enriched in HTLV-1 biofilms and incorporated into the viral envelope. However, we report that only the tetraspanin CD82 interacts with HTLV-1 Gag proteins which initiates their polarization into viral biofilms. Also, we demonstrate that CD82 maintains HTLV-1 biofilm polarization and favors viral transmission, as its silencing induces a complete reorganization of viral clusters at the cell surface and reduces the ability of infected T-cells to transmit the virus. Our results highlight the crucial role of CD82 and its glycosylation state in the architectural organization of HTLV-1 biofilms and their subsequent transfer through intercellular contacts.

**IMPORTANCE:**

In the early stages of infection, human T-lymphotropic virus type 1 (HTLV-1) dissemination within its host is believed to rely mostly on cell-to-cell contacts. Past studies unveiled a novel mechanism of HTLV-1 intercellular transmission based on the remodeling of the host-cell extracellular matrix and the generation of cell-surface viral assemblies whose structure, composition, and function resemble bacterial biofilms. These polarized aggregates of infectious virions, identified as viral biofilms, allow the bulk delivery of viruses to target cells and may help to protect virions from immune attacks. However, viral biofilms’ molecular and functional description is still in its infancy, although it is crucial to fully decipher retrovirus pathogenesis. Here, we explore the function of cellular tetraspanins (CD9, CD81, CD82) that we detect inside HTLV-1 particles within biofilms. Our results demonstrate specific roles for CD82 in the cell-surface distribution and intercellular transmission of HTLV-1 biofilms, which we document as two essential parameters for efficient viral transmission. At last, our findings indicate that N-glycosylation of cell-surface molecules, including CD82, is required for the polarization of HTLV-1 biofilms and for the efficient transmission of HTLV-1 between T-lymphocytes.

## INTRODUCTION

The human T-lymphotropic virus type 1 (HTLV-1) is the most potent oncogenic virus known to date, with a minimal estimation of 5 to 10 million people infected worldwide (1, 2). Although most people remain asymptomatic following HTLV-1 exposure, chronic infections can lead to aggressive pathologies with poor prognoses such as adult T-cell leukemia (3, 4), progressive inflammatory disorders like HTLV-1-associated myelopathy/tropical spastic paraparesis (HAM/TSP) (5, 6), and other less severe pathologies (uveitis, dermatitis, myositis, etc.) (7). *In vivo*, HTLV-1 is primarily detected in CD4(+) T-cells and to a lesser extent in CD8(+) T-cells, monocytes, or dendritic cells. Transmission among individuals occurs through three main routes: mother to child during breastfeeding, sexual contact, and exposure to HTLV-1-infected blood products (8). Overall, this virus represents a major health issue as no therapeutic strategy allows the efficient protection of exposed individuals. Therefore, fundamental research on HTLV-1 is essential to promote the expansion of diagnosis methods and pertinent therapeutic approaches.

HTLV-1 belongs to the *Deltaretrovirus* genus of the Retroviridae family and thus requires the expression of its main structural protein, the Gag polyprotein, to drive particle assembly, budding, and release from the plasma membrane (9). In terms of structure, HTLV-1 is enveloped and contains two single-stranded genomic RNA molecules sheltered by an icosahedral capsid (10). As such, viral particles are mostly spherical and display sizes ranging from 76 to 175 nm in diameter (11, 12). Although HTLV-1 structural details have been uncovered along with its macroscopic transmission routes, HTLV-1 cell-to-cell transmission pathways remain to be completely elucidated. Unlike most retroviruses, evidence strongly suggests that cell-free virions are poorly infectious *in vivo*. This belief originated from transfusion studies showing that, unlike plasma fractions or plasma derivatives where viral RNAs were rarely detected, cellular blood components were strictly needed for productive infections (13, 14). *In vitro*, previous experiments supported this notion and demonstrated that cell-free viruses do not efficiently infect primary T-lymphocytes as compared to co-cultures with HTLV-1-infected cell lines (15, 16).

HTLV-1 cell-to-cell transmission strategies described in models mimicking its preferential tropism [i.e., CD4(+) T cells] include the virological synapse, the cellular conduits, and viral biofilms (for a review, see reference 17). These three pathways, which may not be mutually exclusive, display similarities as they all allow the transfer of viral particles to target cells and involve plasma membrane remodeling. On one hand, the virological synapse is described as a cytoskeleton-dependent, stable adhesive junction that enables direct virus transfer (18). On the other hand, cellular conduits are membrane protrusions that act as an avenue for the movement of viral particles toward target cells (19). At last, viral biofilms are cell-surface aggregates of viruses enriched in carbohydrates, glycoproteins (i.e., agrin), intercellular adhesion molecules (i.e., ICAM-1), and extracellular matrix (ECM) components up-regulated by HTLV-1 (i.e., galectin-3, collagen, fibronectin, sialyl Lewis X) that promote virions accumulation at one or several poles of the infected cells (20–24). These structures allow the bulk delivery of infectious particles to target cells and may help to protect HTLV-1 virions from immune attacks (25, 26). In addition to chronically infected T-cells, HTLV-1 biofilms were also observed at the surface of primary CD4(+) T-cells from HAM/TSP patients or asymptomatic HTLV-1 carriers (24). While the relative importance of the different cell-to-cell transmission routes is difficult to ascertain *in vivo*, the removal of biofilms by heparin washes reduces the infectious capacity of HTLV-1-producing cells by 80% *in vitro* (24). In addition, biofilms detached from HTLV-1-infected cells are infectious *in vitro,* as opposed to cell-free virions released in the supernatant of the same cells (16, 24), indicating that the transfer of HTLV-1 biofilms through cell-to-cell contacts is the most efficient pathway to infect new cells.

To identify molecular actors involved in HTLV-1 biofilm architecture and transmission, we focused here on the role of specific biofilm components belonging to the transmembrane 4 superfamily of proteins, also known as tetraspanins. Tetraspanins are four-span transmembrane proteins organized in tetraspanin-enriched microdomains (TEMs) at the plasma membrane, where they self-associate through their large extracellular loops or interact with various protein complexes, thereby generating a hierarchical network of interactions (for a review, see reference 27). They are expressed by all metazoans, with 33 members in mammals, including CD9, CD63, CD81, CD82, and CD151. Among their known functions, tetraspanins regulate T-cell adhesion (cell-to-cell and cell-to-ECM), synapse formation, cell motility, and membrane compartmentalization. In addition, several pathogens hijack these surface proteins for infection, with CD81 being the main receptor of hepatitis C virus entry for instance, or CD151 being involved in human papillomavirus endocytosis (28, 29). Concerning retroviruses, it has been shown for the human immunodeficiency virus 1 (HIV-1) that viral particles accumulate in membrane microdomains enriched in CD63, CD81, and CD82 in infected cells (30–32). In addition, CD81, which is found in the viral membrane, is required for the polarization of HIV-1 Gag clusters at the T-cell surface (30). For HTLV-1, Mazurov and collaborators demonstrated that CD9, CD53, CD63, and CD82 are found in viral aggregates at the surface of chronically infected T-cells, although no function was addressed in their study. Nevertheless, it was shown that HTLV-1 Gag can form intracellular complexes with CD81 and CD82 intracellular loops when over-expressed in human embryonic kidney 293 (HEK293) cells (21, 33). Another study performed with chronically infected T-cells reported the interaction between CD82 and HTLV-1 envelope glycoprotein (Env) which remains associated during their trafficking to the plasma membrane through the secretory pathway (34). Overall, these observations suggest a function for cellular tetraspanins in viral protein trafficking, viral assembly, and/or biofilm formation, which could subsequently contribute to HTLV-1 cell-to-cell transmission.

Here, we asked whether these tetraspanins could be incorporated into viral particles within HTLV-1 biofilms, participate in biofilm biogenesis at the T-cell surface, and play a role in the subsequent transmission of HTLV-1 biofilms to target T-cells. In our study, we show the enrichment of CD9, CD81, and CD82 in HTLV-1 biofilms released from infected T-cells using mass spectrometry coupled to immunoblotting, along with the strong polarization of these three transmembrane molecules into cell-attached biofilms using immunofluorescence microscopy. Accordingly, we demonstrate the incorporation of CD9, CD81, and CD82 in the viral lipidic envelope using super-resolution stimulated emission depletion (STED) microscopy and immuno-electron microscopy (IEM). At last, using shRNAs to silence the expression of CD9, CD81, or CD82 in chronically infected T-cells, we identify a crucial role for CD82 in the regulation of HTLV-1 biofilm polarization at the cell surface. Its silencing depolarizes HTLV-1 Env(+) aggregates and reduces the ability of HTLV-1-producing cells to infect target T-cells. While the specific mechanisms underlying CD82-dependent polarization of HTLV-1 biofilms remain to be elucidated, our findings indicate a potential involvement of its N-glycosylation chains. Indeed, we demonstrate that N-glycosylation of cell-surface molecules, including CD82, is essential for the polarization and infectivity of HTLV-1 biofilms. Overall, our results highlight a specific role for the tetraspanin CD82 in HTLV-1 clustering and biofilm polarization, which are essential parameters for efficient viral intercellular transmission.

## RESULTS

### Super-resolution microscopy imaging confirms the integrity of HTLV-1 biofilms isolated from infected T-cells

**Fig 1:**
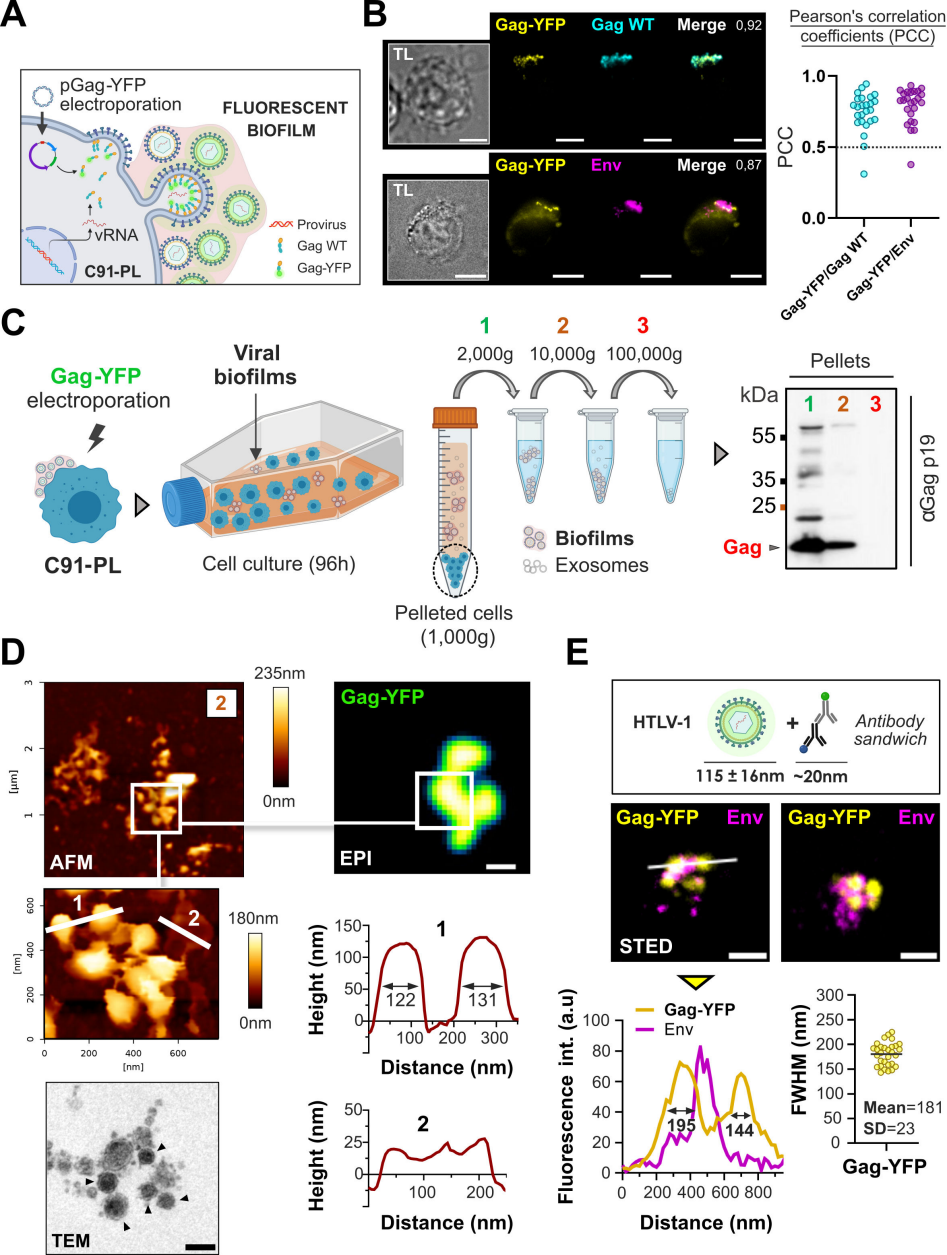
Super-resolution microscopy imaging confirms the integrity of HTLV-1 biofilms isolated from infected T-cells. (**A**) Scheme: electroporation of HTLV-1 chronically infected T-cells (**C91-PL**) with a plasmid encoding HTLV-1 Gag-YFP. (**B**) Left: representative images showing the successful incorporation of Gag-YFP (yellow) into Gag(+) (cyan) and Env(+) (magenta) virions. TL, transmitted light. Right: Pearson’s correlation coefficients as a measure of colocalization between Gag-YFP/Gag WT and Gag-YFP/Env (*n* = 25 cells in each condition): this coefficient describes the linear relationship between the gray levels of the two channels : 0 = no correlation; 1 = 100% correlation between both channels. Scale bars = 10 µm. (**C**) Left: protocol for isolation of fluorescent biofilms from chronically infected T-cells (C91-PL). Right: immunoblot probed for HTLV-1 Gagp19 showing Gag expression in biofilms isolated at 2,000 g (1), 10,000 g (2), or 100,000 g (3). (**D**) Top: atomic force microscopy correlated to fluorescence imaging of native Gag-YFP(+) biofilms pelleted at 10,000 g (2). Gag-YFP(+) structures (biofilms) revealed by wide-field fluorescence microscopy were imaged by atomic force microscope (topographic images). Higher magnification shows spherical particles whose size corresponds to HTLV-1 diameter (FWHM on the first cross-section/upper plot = 122 nm and 131 nm) and thin structures bound to the particles (second cross-section/lower plot). Scale bar = 500 nm. Bottom left: transmission electron microscopy image of cell-free HTLV-1 biofilm. Individual viral particles are indicated with black arrows. Scale bar = 200 nm. (**E**) Top: scheme of HTLV-1 Gag-YFP(+) particles’ expected size after staining. Middle: representative STED microscopy images of fixed Gag-YFP(+) biofilms pelleted at 10,000 g and stained for the viral envelope protein (Env). Scale bars = 500 nm. Bottom: the white cross-section is plotted below the corresponding image (left graph) where the two yellow peaks correspond to individual particles (FWHM = 195 nm and 144 nm). The right plot shows the FWHM distribution of *n* = 25 Gag-YFP(+) particles. FWHM, full width at half maximum; SD, standard deviation of the mean.

**Fig 2:**
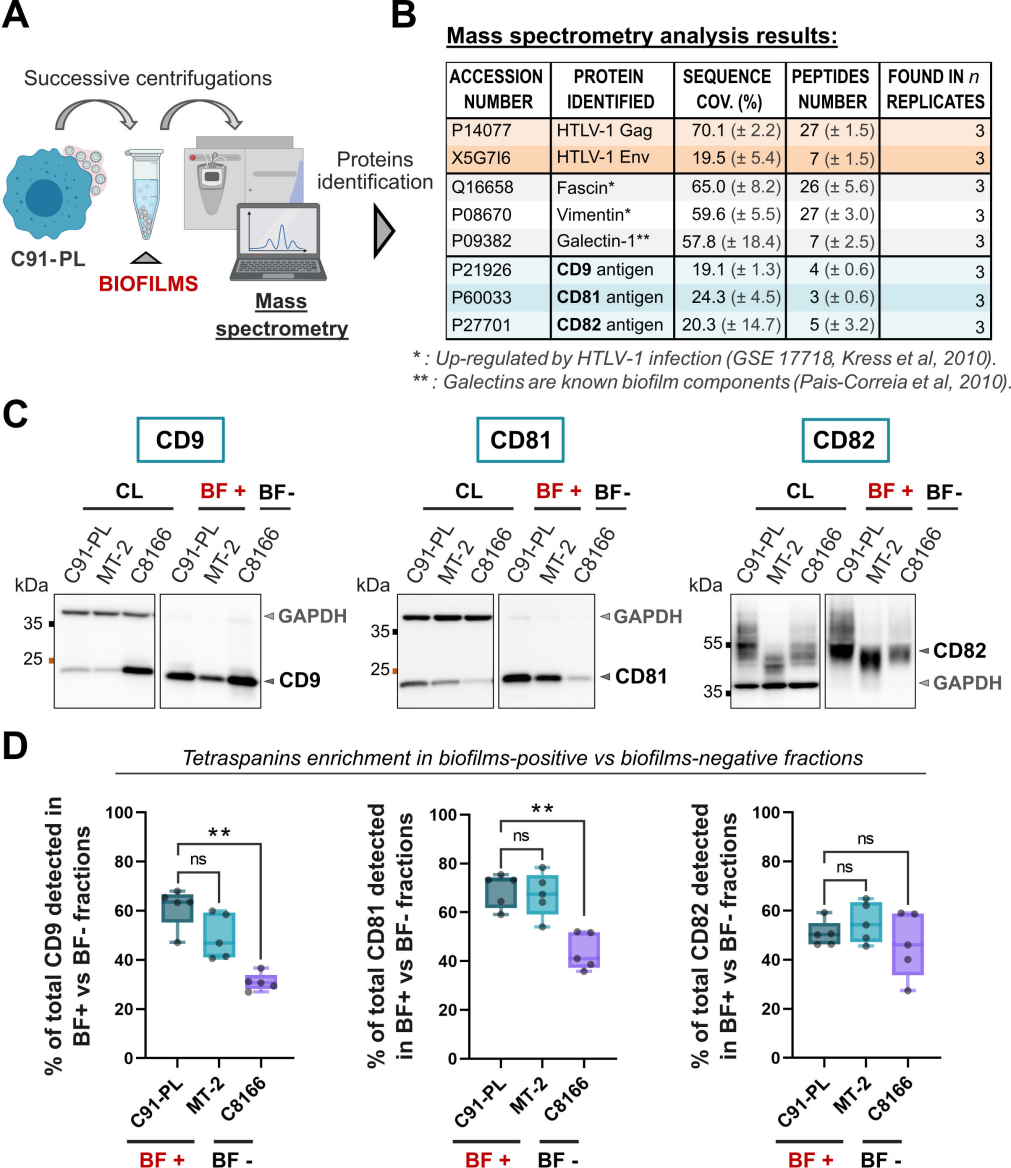
CD9, CD81, and CD82 are detected and enriched in viral biofilms released from HTLV-1 chronically infected T-cells. (**A**) Summary of the protocol used to isolate wild-type biofilms released in the cell culture medium of HTLV-1 chronically infected T-cells (C91-PL). This protocol is detailed in Fig. 1C. (**B**) Table gathering a subset of proteins identified with high confidence by mass spectrometry (MS) in biofilms isolated from HTLV-1-infected T-cells (C91-PL). A more detailed list of proteins identified consistently by MS is provided in Table S2, and a STRING interaction network of the 100 most abundant proteins is given in Fig. S2. CD9, CD81, and CD82 tetraspanins (blue) were identified in isolated biofilms along with viral proteins (orange) and cellular proteins (gray) known to be upregulated by HTLV-1 infection (*) or to be present in biofilms (**). Results were obtained from three independent experiments; standard deviation values are given in brackets. (**C**) Immunoblots of CD9, CD81, and CD82 contained in cell lysates (CL); biofilms-positive supernatants (BF+); or biofilms-negative supernatants (BF−) from three different cell lines: two cell lines infected by HTLV-1 that produce biofilms (BF+: C91-PL, MT-2), and one cell line infected by HTLV-1 that is deficient in virus production (BF−: C8166). Endogenous GAPDH is used as a loading control for cell lysates. (**D**) Tetraspanin release in biofilms was calculated as the ratio of the amount of tetraspanins in BF [corrected by the dilution factor (DF)] to the total amount of tetraspanins (CL + BF) following this formula: [(BF * DF)/(BF * DF + CL * DF)] * 100. Box plots show the corresponding results for CD9, CD81, and CD82 released by each cell line in %. *n* = 5 independent experiments. Kruskal-Wallis statistical tests were used to assess any significant difference: ns = *P*-value >0.05; ** = *P*-value ≤0.01, as compared to the C91-PL.

Viral biofilms isolated from HTLV-1-infected T-cells are known to be infectious units as they can productively infect primary T-cells and monocyte-derived dendritic cells *in vitro* (16). Thus, they are relevant minimal systems to study molecular components linked to HTLV-1 cell-to-cell transmission. In the present study, we produced and purified non-fluorescent and fluorescent HTLV-1 biofilms to further investigate their structural (Fig. 1) and molecular composition (Fig. 2).

To first decipher the structure and morphology of HTLV-1 biofilms, C91-PL chronically infected T-cells, which produce large and polarized viral biofilms, were electroporated with a plasmid encoding the fluorescent viral protein Gag-YFP (Fig. 1A). Of note, the fusion of the YFP tag at the C-terminal end of Gag was shown to not impair its trafficking or pseudo-particle assembly (35). Twenty hours post-electroporation, living Gag-YFP(+) infected cells started to exhibit viral aggregates that accumulated within a few hours into large, polarized biofilms following lateral movements of preformed Gag-YFP(+) clusters toward intercellular contacts (Video S1; Fig. S1). These Gag-YFP(+) clusters colocalized extensively with endogenous Gag and Env viral proteins, as shown by their respective Pearson’s correlation coefficients (PCC) of 0.75 on average for Gag-YFP/Gag WT and of 0.79 on average for Gag-YFP/Env (Fig. 1B). This confirms that Gag-YFP fluorescent signal reflects nascent virion formation and accumulation in biofilms. We then maintained Gag-YFP(+) C91-PL cells at a high cellular density to promote the release of biofilms in the culture supernatant that were collected using sequential centrifugations (2,000 *× g*, 10,000 *× g*, and 100,000 *× g*), as shown in Fig. 1C. While cell-free enveloped viruses are usually sedimented by >100,000 *× g* ultracentrifugation (36), most HTLV-1 particles detected using Gag-p19 immunoblotting were sedimented at 10,000 *× g*, suggesting their nearly complete retention in dense structures corresponding to viral biofilms (Fig. 1C). Based on these observations, we always harvested HTLV-1 biofilms at 10,000 *× g* throughout the study.

To assess whether intact HTLV-1 biofilms were successfully isolated using this method, we imaged our samples using an atomic force microscope (AFM) coupled with fluorescence microscopy (37), which allowed us to distinguish individual virions (Fig. 1D). As shown in the magnified AFM image in Fig. 1D, Gag-YFP(+) biofilms contained spherical particles with a mean central height of 126 nm  ± 8 nm (e.g., first cross-section). This particle size is consistent with the diameter of HTLV-1 virions measured by cryogenic transmission electron microscopy (11, 12) (i.e., 115 nm ±16 nm, with a broad size distribution ranging from 76 nm to 175 nm). Moreover, Gag-YFP(+) viral particles appeared interconnected by a network of fine filamentous structures (Fig. 1D) that exhibited a mean height of 29 nm  ± 6 nm (e.g., second cross-section). Although these structures are unlikely to be plasma membrane extensions since they are five times thicker than model lipid bilayers (38), they could nonetheless correspond to scaffolds of ECM components. This hypothesis is supported by transmission electron microscopy images of cell-free HTLV-1 biofilms where viral particles are associated with electron-dense ECM structures (Fig. 1D).

Following the same isolation protocol, we explored if these fluorescent particles successfully incorporated the Env glycoprotein in their membrane using super-resolution STED microscopy, which allows us to go below 100 nm in x, y resolution (for a review, see reference 39). Most Gag-YFP(+) particles (full width at half maximum = 181 nm  ± 23 nm) were positive for Env and clustered together in biofilms (Fig. 1E). Although some particles did not exhibit Env signals at their surface, it is known that HTLV-1 particles contain variable numbers of Env molecules, most of which are unevenly distributed in the viral lipidic envelope (40). Overall, our results demonstrate the successful isolation of HTLV-1 biofilms containing unaltered Gag(+)/Env(+) viral particles.

### HTLV-1 biofilms released from infected T-cells are enriched in tetraspanins

To screen for key molecular components of HTLV-1 biofilms, we performed a large-scale identification of viral and cellular proteins contained in isolated biofilms using mass spectrometry. Biofilms produced from C91-PL chronically infected cells were isolated as in Fig. 1C, inactivated, and processed on a mass spectrometer (Fig. 2A). Among all the proteins identified, we excluded contaminants (including mitochondrial and ribosomal proteins) and considered only the 100 most abundant proteins (based on their intensity based absolute quantification IBAQ score) that were identified with at least three peptides in three independent replicates. For a detailed list that includes less abundant proteins, see Table S2. As expected, viral proteins (Gag, Env) were detected in C91-PL biofilms (Fig. 2B), along with proteins up-regulated by HTLV-1 infection (e.g., fascin [41], vimentin, GSE17718 database [42]) and extracellular matrix proteins like galectins (43), which are known components of HTLV-1 biofilms (24) (Fig. 2B). Thus, the presence of HTLV-1-associated proteins validated the successful isolation of viral biofilms, allowing the reliable identification of new biofilm components.

Among the 100 most abundant proteins detected in isolated biofilms, 69 were found to be involved in protein-protein interaction networks related to the cytoskeleton or the cellular surface (Fig. S2). Importantly, a cluster of three tetraspanins was identified with high confidence in HTLV-1 biofilms: CD9, CD81, and CD82 (Fig. 2B). To further determine whether CD9, CD81, and CD82 were enriched in HTLV-1 biofilms, we quantified their release in the supernatant of three different cell lines (Fig. 2C): (i) C91-PL and (ii) MT-2 chronically infected T-cells that both produce viral particles and biofilms [“Biofilms-positive,” BF(+)], or (iii) C8166-infected T-cells that do not express Env/Gag structural proteins (44) and are thus deficient in virus production [“Biofilms-negative,” BF(−)]. Tetraspanins release was calculated as the ratio of the amount of tetraspanins in BF(+) fractions (isolated from C91-PL/MT-2 cells) or BF(−) fractions (isolated from C8166 cells) to the total amount of tetraspanins (see “Western blot analysis” in Materials and Methods). As shown in Fig. 2D, around 60% of total CD9 was detected in BF(+) fractions from C91-PL/MT-2 cells against less than 35% in BF(−) fractions from C8166 cells. A similar tendency was observed for CD81 which was significantly enriched in BF(+) as compared to BF(−) fractions (Fig. 2D). At last, we observed no significant difference between the amount of CD82 retrieved in BF(+) and BF(−) fractions (Fig. 2D). Overall, our results demonstrate that CD9, CD81, and CD82 are consistently detected in HTLV-1 biofilms and that CD9 and CD81, but not CD82, are significantly enriched in biofilms released from virus-producing T-cells.

### CD9, CD81, and CD82 are polarized in biofilms at the surface of HTLV-1-producing T-cells

**Fig 3:**
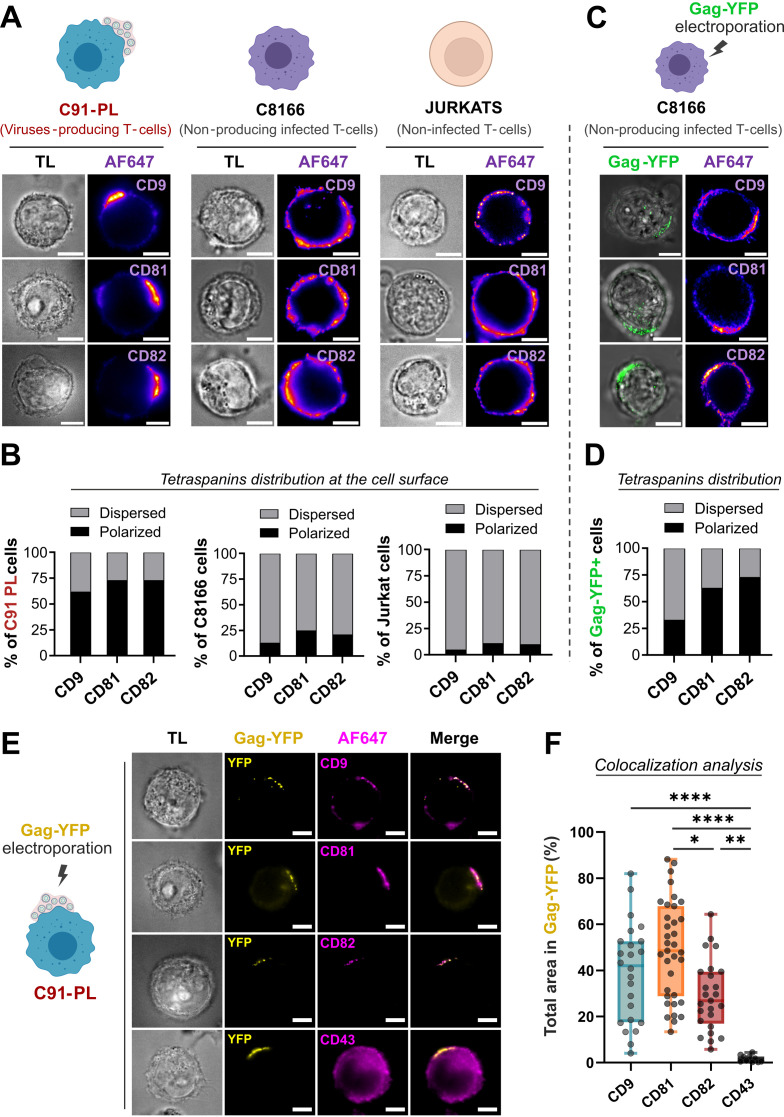
CD9, CD81, and CD82 are polarized in viral biofilms at the surface of HTLV-1-producing T-cells. (**A**) Representative confocal microscopy images showing CD9, CD81, and CD82 tetraspanin localization at the surface of fixed non-permeabilized cells. Each image is a projection of 10 successive optical z-slices of 1 µm. Two major patterns were observed in HTLV-1 chronically infected C91-PL cells (in red) and control cells (C8166 and Jurkat): “polarized”*—*tetraspanins are clustered on one side of the cell surface, or “dispersed”—tetraspanin signal is distributed all over the cell periphery as punctuated small dots. Representative images of the most abundant pattern (“polarized” or “dispersed”) observed in each condition are shown. TL, transmitted light. Scale bars = 10 µm. (**B**) Quantification of CD9, CD81, and CD82 distribution patterns in the cell population for each cell line (*n* > 30 cells counted for each condition). On the histograms, gray bars = % of cells displaying the dispersed pattern; black bars = % of cells displaying the polarized pattern for each tetraspanin tested. (**C**) Representative confocal microscopy images of tetraspanin localization at the surface of C8166 cells upon transient Gag-YFP expression. Each image is a projection of 10 successive optical z-slices of 1 µm. Scale bars = 10 µm. (**D**) CD9, CD81, and CD82 distribution patterns in the cell population were quantified as in (**B**) (*n* > 30 cells counted for each condition) and reported on the histogram. (**E**) C91-PL cells (including the ones presented in Fig. 3A) were electroporated with Gag-YFP and stained for CD9, CD81, CD82, or CD43. Representative confocal microscopy images of Gag-YFP(+) C91-PL cells showing the localization of CD9, CD81, CD82, or CD43 (magenta) along with Gag-YFP(+) biofilms at the cell surface (yellow) (including one duplicated cell of Fig. 3A). TL, transmitted light. Scale bars = 10 µm. (**F**) Colocalization analysis: box plot showing the total area of CD9, CD81, CD82, or CD43 signals (channel 2) overlapping Gag-YFP(+) biofilms (channel 1) in %, quantified for each condition (*n* > 25 cells). See Fig. S3C for thresholding and calculation details. Kruskal-Wallis statistical tests were used to assess any significant difference: * = *P*-value ≤0.05; ** = *P*-value ≤0.01; **** = *P*-value ≤0.0001.

Following the detection of CD9, CD81, and CD82 in released HTLV-1 biofilms, we examined their localization at the surface of chronically infected T-cells (C91-PL), in comparison with infected T-cells deficient in virus production (C8166) or with non-infected T-cells (Jurkat). Accordingly, fixed C91-PL, C8166, and Jurkat cells were immuno-stained for CD9, CD81, or CD82 and imaged using confocal microscopy (Fig. 3A). Two major phenotypes emerged for the three molecules: a “polarized” pattern where tetraspanins were clustered on one side of the cell surface, mainly observed in HTLV-1-producing cells (C91-PL); and a “dispersed” pattern where their signal was distributed all over the cell periphery, mainly observed in infected T-cells deficient in virus production (C8166) and in non-infected control cells (Jurkat) (see Fig. S3A for a more detailed image gallery). We quantified the occurrence of these phenotypes per cell line in Fig. 3B. While 70% to 90% of control cells displayed a dispersed pattern for all tetraspanins tested, this phenotype was the opposite in C91-PL cells (Fig. 3B), where at least 70% of cells carried polarized aggregates of CD9, CD81, and CD82. To check if viral assembly was the minimal requirement to cluster CD9, CD81, and CD82 at the cell surface, C8166 cells were electroporated with HTLV-1 Gag-YFP constructs (Fig. 3C), knowing that Gag is the minimal system for pseudo-viral particle assembly and release (35). This resulted in a small increase of cells displaying a polarized pattern for CD9 (from 13% to 33%), and a completely reversed phenotype for both CD81 and CD82 that were found polarized in more than 60% of Gag-YFP(+) C8166 cells (Fig. 3D, see Fig. S3B for a more detailed image gallery). This indicates that (i) HTLV-1 Gag is required but not sufficient to induce an extensive CD9 polarization in cells lacking other viral assembly components (i.e, Env), whereas (ii) Gag expression almost completely restores the polarized pattern observed for CD81 and CD82 in C91-PL cells.

To investigate whether these polarized tetraspanins were associated with HTLV-1 biofilms at the cell surface, we used chronically infected T-cells (C91-PL) electroporated with Gag-YFP constructs (schematized in Fig. 1A). Gag-YFP(+) T-cells were fixed and stained for CD9, CD81, CD82, or control CD43. CD43 is a transmembrane glycoprotein reported to be distributed all around the cell periphery without being specifically enriched in HTLV-1 biofilms (45) and is therefore used here as a negative control. As expected, confocal images showed no colocalization between Gag-YFP(+) biofilms and CD43 (Fig. 3E). However, an extensive colocalization was observed between CD9, CD81, or CD82 and Gag-YFP(+) biofilms that were polarized together on one side of the cell surface (Fig. 3E). To quantify the degree of colocalization, we calculated the percentage of the total area occupied by each tetraspanin that was overlapping with Gag-YFP(+) biofilms (see Fig. S3C for calculation details). While the percentage of total CD43 area overlapping with Gag-YFP(+) signals was negligible (around 2%), 50% of the total area occupied by CD9 or CD81 colocalized with Gag-YFP(+) biofilms (Fig. 3F). A milder but still significant colocalization was observed for CD82, with approximately 30% of its total area polarized in Gag-YFP(+) biofilms. Altogether, these results highlight that Gag expression is sufficient to initiate tetraspanin polarization and that CD9, CD81, and CD82 concentrate in HTLV-1 biofilms at the surface of infected T-cells.

### CD9, CD81, and CD82 are incorporated into individual HTLV-1 particles

As we showed that tetraspanins were released within HTLV-1 biofilms and clustered in their vicinity, we now examined if CD9, CD81, and CD82 were incorporated into the virus envelope. To this aim, we used STED microscopy and IEM which provide super-resolution imaging to visualize and discriminate individual virions. As we showed in Fig. 1E using isolated biofilms, the diameter of individual Gag-YFP(+) virions measured by STED microscopy is 181 nm ± 23 nm.

**Fig 4:**
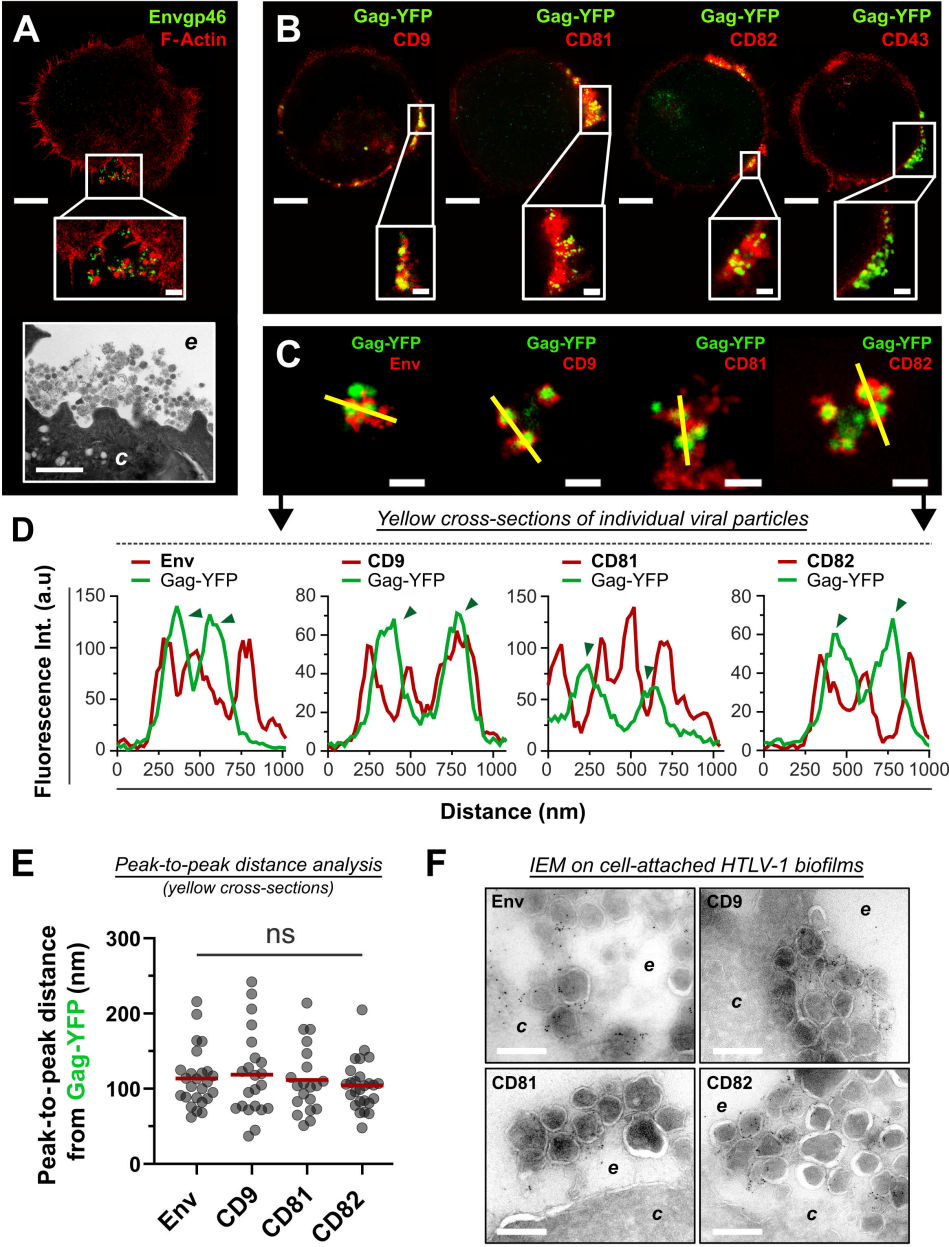
CD9, CD81, and CD82 are incorporated into individual HTLV-1 particles. (**A**) Top: representative STED 2D image of an HTLV-1-infected T-cell (C91-PL) permeabilized and stained for cortical F-actin (red) and for HTLV-1 viral envelope (green). Scale bar = 5 µm. Middle: higher magnification of the Env(+) viral biofilm. Scale bar = 500 nm. Bottom: transmission electron microscopy image of HTLV-1 biofilm at the surface of a C91-PL cell. Scale bar = 1 µm. *c*, cytosol; *e*, extracellular environment. (**B**) Top: representative STED 2D images showing Gag-YFP(+) viruses embedded in CD9, CD81, or CD82 enriched domains at the surface of C91-PL cells. Scale bars = 5 µm. Bottom: zoomed images of viral biofilms. Scale bars = 500 nm. (**C**) Representative STED 2D images of Gag-YFP(+) cell-free biofilms (isolated as detailed in Fig. 1C) stained for Envgp46, CD9, CD81, or CD82 (red). Scale bars = 500 nm. (**D**) Graphs corresponding to the yellow cross-sections in panel C. Arrows indicate individual particles (FWHM ≈ 150–200 nm). (**E**) Scatter plot showing the distribution of the distances measured between the peak centers of Env/CD9/CD81/CD82 signals (extracted from panel C cross-sections) and the peak centers of Gag-YFP signals (*n* > 20 peak-to-peak distances measured for each condition). Mean = red bar. Kruskal-Wallis statistical tests were used to assess any significant difference: ns, nonsignificant-significant. (**F**) Immuno-electron microscopy images showing viral biofilms at the surface of C91-PL cells stained for Env, CD9, CD81, or CD82 with antibodies coupled to 6 nm gold beads. *c*, cytosol ; *e*, extracellular environment. Scale bars = 200 nm. FWHM, full width at half maximum.

First, C91-PL cells were stained for cortical F-actin, which highlights cell membrane boundaries, and for HTLV-1 Env to identify virions (Fig. 4A). Viral biofilms appeared as dense structures enriched in mature virions (Env-positive) that were found at the surface of infected cells without being necessarily associated with the host cell plasma membrane (see Fig. S4A for more acquisitions). This was confirmed by transmission electron microscopy imaging of HTLV-1-infected T-cells (Fig. 4A) where viral biofilms concentrate virions and ECM components on top of the plasma membrane (see Fig. S4B for more acquisitions). To test whether tetraspanins were similarly detected inside released particles accumulated in biofilms, C91-PL cells were electroporated with Gag-YFP and stained for CD9, CD81, CD82, or CD43 (Fig. 4B). As opposed to CD43 which was only found at the plasma membrane and seemed to be excluded from viral aggregates, individual HTLV-1 particles were embedded in dense networks of CD9, CD81, and CD82 (magnified images in Fig. 4B). This suggests that in addition to the plasma membrane, CD9, CD81, and CD82 also accumulate in the periphery of HTLV-1 particles. However, we could not exclude at this point that tetraspanins could be trapped in membrane protrusions within biofilms and not directly incorporated into HTLV-1 particles. To discriminate between these two hypotheses, Gag-YFP(+) biofilms were isolated as in Fig. 1C, stained for Env, CD9, CD81, or CD82, and imaged using STED microscopy (Fig. 4C). The three tetraspanins were observed at the periphery of isolated Gag-YFP(+) particles in an Env-like pattern, without being detected in the particles’ core (positive for Gag-YFP but negative for CD9, CD81, or CD82, Fig. 4C and D). Moreover, we measured an equivalent distance between Env-Gag signals and Gag-tetraspanin signals using peak-to-peak distances analysis on cross-sections from Fig. 4D. On average: Env-Gag distance = 114 nm ± 39 nm; CD9-Gag distance = 119 nm ±56 nm; CD81-Gag distance = 112 nm ±45 nm; and CD82-Gag distance = 105 nm ±33 nm (Fig. 4E). These results indicate that Env, CD9, CD81, and CD82 are equidistant from the particles’ center, suggesting that tetraspanins are incorporated into the viral envelope. This was confirmed by IEM (Fig. 4F) where Env, CD9, CD81, and CD82 were all detected at the periphery of HTLV-1 particles contained in biofilms at the surface of C91-PL cells (see Fig. S4C for larger magnifications). While tetraspanins accumulated in HTLV-1 virions, we could not detect any signal for CD9, CD81, or CD82 at the plasma membrane by IEM suggesting a specific enrichment of these molecules in viral particles (Fig. 4F). Altogether, our findings highlight the Env-like incorporation of CD9, CD81, and CD82 into viral particles aggregated within isolated or cell-surface HTLV-1 biofilms.

### CD82 regulates the polarization of HTLV-1 biofilms at the surface of infected T-cells

**Fig 5:**
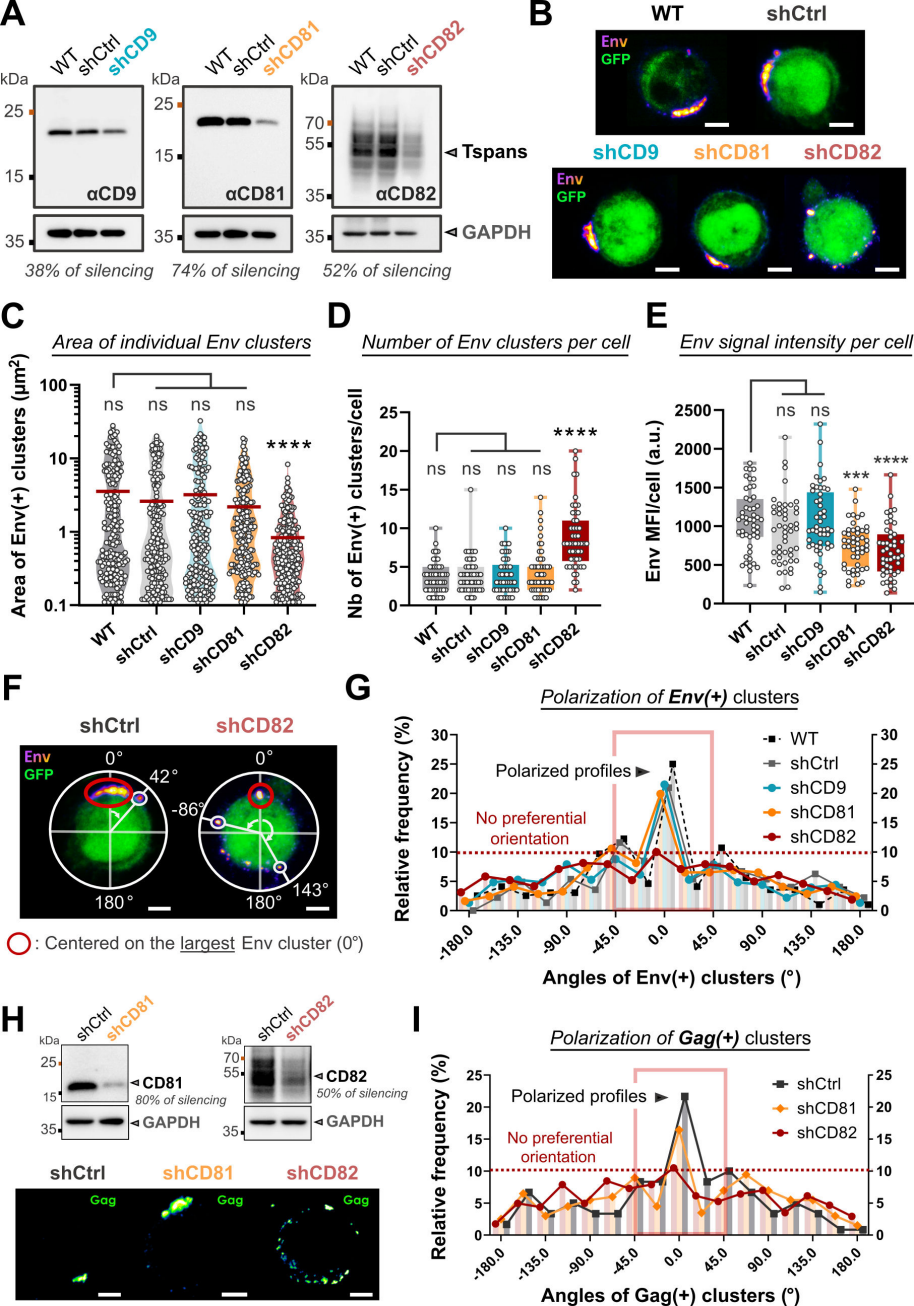
CD82 silencing induces the depolarization of HTLV-1 biofilms at the surface of infected T-cells. (**A**) Immunoblots showing CD9, CD81, and CD82 expression in C91-PL cells transduced or not (WT) with scrambled shRNAs (shCtrl) or shRNAs targeting CD9, CD81, or CD82, 5 days post-transduction. Endogenous GAPDH is used as a loading control. Silencing efficiency is reported below each immunoblot and was calculated as the ratio between the intensity of tetraspanin signals upon silencing and their expression in WT conditions (both normalized to GAPDH). (**B**) Representative confocal images of C91-PL transduced or not (WT, GFP−) with different shRNAs (GFP+)—shCtrl, shCD9, shCD81, or shCD82. All cells were fixed and stained for HTLV-1 Env (fire red) 5 days after shRNAs transduction. Scale bars = 5 µm. (**C**) Violin plot showing the area (µm^2^) of individual Env(+) clusters in control cells versus CD9/CD81/CD82-silenced cells (*n* > 180 clusters per condition). Mean = red bar. (**D**) Box plot showing the number of Env(+) clusters at the surface of control cells versus CD9/CD81/CD82-silenced cells (*n* > 50 cells in each condition). (**E**) Box plot showing the mean fluorescence intensity (MFI) of Env signal per cell in control cells versus CD9/CD81/CD82-silenced cells (*n* > 50 cells). (**F**) Representative confocal images showing examples of the analysis performed to assess the angular distribution of HTLV-1 clusters at the cell surface (see “Confocal image analysis” in Materials and Methods). (**G**) Frequency distribution of the angles of Env(+) clusters at the surface of control cells (WT, shCtrl) versus CD9/CD81/CD82-silenced cells (*n* > 180 clusters for *n* > 50 cells per condition). While a peak between −45° and 45° (red square) is representative of a polarized profile, the absence of a peak indicates a depolarized pattern (red dotted line). (**H**) Top: immunoblots showing CD81 and CD82 expression in C91-PL cells transduced with scrambled shRNAs (shCtrl) or shRNAs targeting CD81 or CD82, 5 days post-transduction. Endogenous GAPDH is used as a loading control. Bottom: representative confocal images of C91-PL transduced with different shRNAs—shCtrl, shCD81, or shCD82. All cells were fixed and stained for HTLV-1 Gag (green fire blue) 5 days after shRNAs transduction. Scale bars = 5 µm. (**I**) Frequency distribution of the angles of Gag(+) clusters at the surface of control cells (shCtrl) versus CD81/CD82-silenced cells (*n* > 120 clusters for *n* > 30 cells per condition). Kruskal-Wallis statistical tests were used to assess any significant difference: ns = *P*-value >0.05; *** = *P*-value ≤0.001; **** = *P*-value ≤0.0001.

After having established the incorporation of CD9, CD81, and CD82 into viral particles within biofilms, we examined their potent functions in the organization of HTLV-1 biofilms at the cell surface, as tetraspanins are known to be molecular scaffolds that distribute partner proteins into highly organized microdomains (for a review, see references 46, 47). To this aim, we designed shRNAs delivered using green fluorescent protein (GFP) positive lentivectors targeting each tetraspanin of interest to silence their expression in chronically infected T-cells (C91-PL). Non-transduced cells (wild-type, WT) along with cells transduced with scrambled shRNAs (shCtrl) were used as negative controls. The efficiency of shRNA silencing in the cell population was controlled by western blot at day 5 post-transduction and reached repeatedly around 40% for shCD9, 70% for shCD81, and 50% for shCD82 (Fig. 5A). Simultaneously, C91-PL cells were fixed and surface-stained for Env at day 5 post-transduction (Fig. 5B). Silenced cells were identified by GFP expression, which was absent in WT C91-PL cells as expected (shown in representative images of Fig. 5B). CD9 and CD81 silencing did not seem to affect the integrity of viral biofilms which remained large, polarized Env(+) aggregates similar to those observed in control cells (Fig. 5B). However, CD82 silencing led to a complete reorganization of HTLV-1 biofilms, with smaller Env(+) clusters scattered all around the cell surface (Fig. 5B). This clear redistribution was also observed when HTLV-1 biofilms were stained for Gag in CD82-silenced C91-PL cells (Fig. 5H).

Accordingly, CD82 silencing resulted in a significant decrease in the area of individual Env(+) clusters (mean area = 1.1 µm^2^), as opposed to CD9 or CD81 silencing (mean area = 2.90 µm^2^, Fig. 5C). This was combined with a significant increase in the number of Env(+) clusters per cell, from a mean of four clusters per cell in controls to a mean of nine clusters per cell in CD82-silenced cells (Fig. 5D). In addition, Env mean fluorescence intensity (MFI) measured at the cell surface of non-permeabilized C91-PL cells showed a 30% decrease upon CD81 and CD82 silencing as compared to the other conditions (Fig. 5E). This suggests that CD81 and CD82 silencing might partially impair Env recruitment to the plasma and/or virion accumulation at the cell surface.

Next, we examined the distribution profiles of Env(+) clusters at the surface of infected T-cells (C91-PL) to quantify HTLV-1 biofilm reorganization upon CD9, CD81, or CD82 silencing as exemplified in Fig. 5F (see “Confocal image analysis” in the Materials and Methods section for analysis details). For WT, shCtrl, shCD9, and shCD81 conditions, around 45% of all Env(+) clusters were detected close to 0° (which is positioned on the largest cluster), indicating a preferential orientation at the cell surface (“polarized profile,” Fig. 5G). However, upon CD82 silencing, Env(+) clusters were redistributed at the cell surface with an increased number of viral aggregates further away from 0° and less than 30% between −45° and 45°, which indicates a depolarized pattern (“no preferential orientation,” Fig. 5G). With a similar behavior observed for Gag(+) clusters upon CD82 silencing (Fig. 5I), we examined if endogenous CD82 was physically interacting with HTLV-1 structural proteins by performing coimmunoprecipitation assays using chronically infected T-cells (Fig. S5). Although we did not detect any interaction between Gag and CD9 or CD81, our results show that HTLV-1 Gag repeatedly co-immunoprecipitated with CD82, which demonstrates their ability to form complexes in infected T-cells (Fig. S5). Overall, our results demonstrate that tetraspanins play a critical role in the accumulation of virions at the cell surface and that CD82 is required to maintain HTLV-1 biofilm polarization, most probably through interactions with Gag.

### CD82 is required for efficient HTLV-1 cell-to-cell transmission

**Fig 6:**
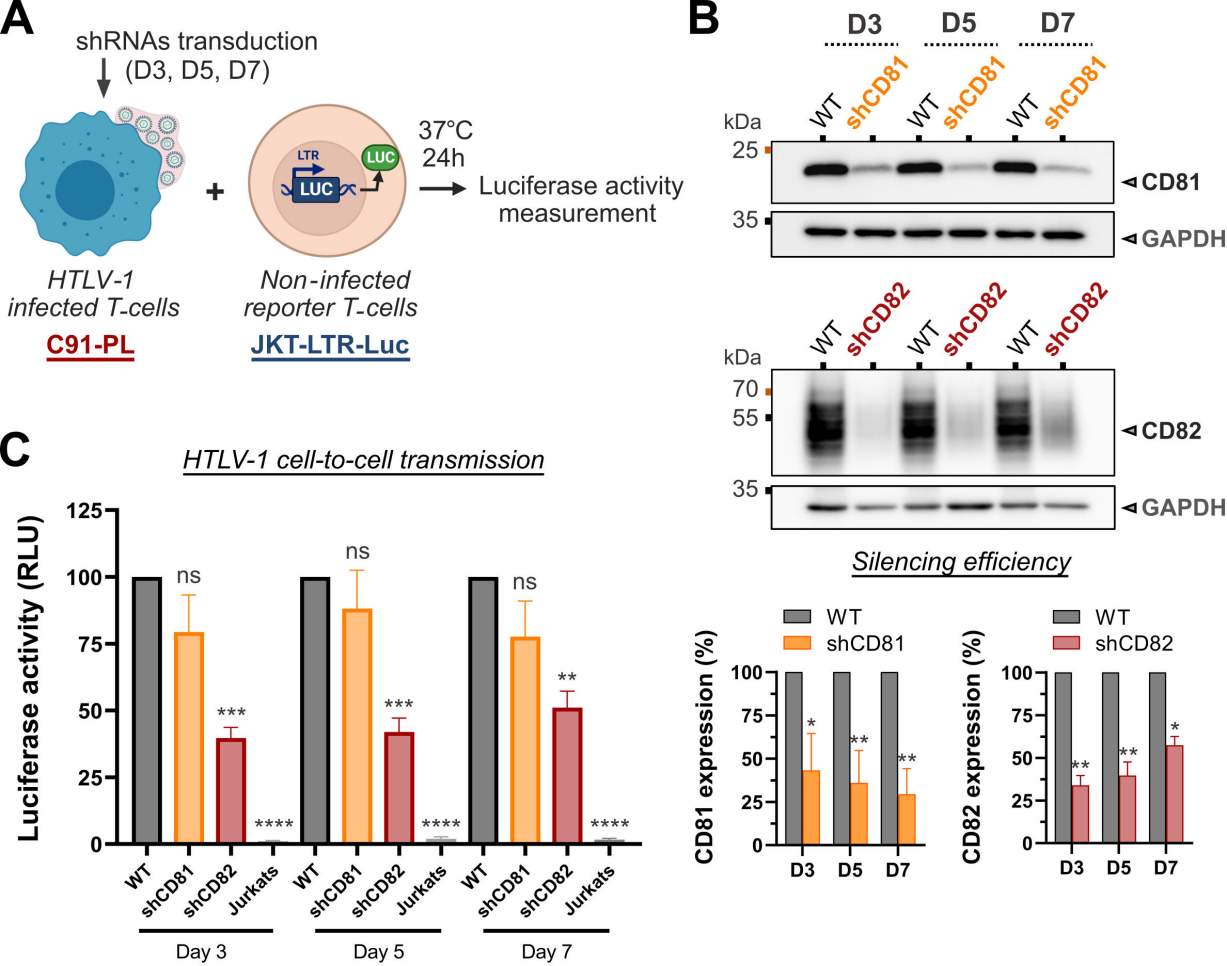
CD82 is required for efficient HTLV-1 cell-to-cell transmission. (**A**) Luciferase reporter assay scheme: C91-PL cells transduced or not with shRNAs targeting CD81 or CD82 for 3, 5, or 7 days, were co-cultured with non-infected reporter Jurkat T-cells for 24 hours. Reporter cells are stably transfected with a plasmid bearing the luciferase gene downstream of the HTLV-1 LTR promoter which is transactivated upon infection by the viral transactivator protein Tax. (**B**) Western blots showing CD81 or CD82 expression in shRNA-transduced C91-PL cells versus control cells (WT: non-transduced cells). Endogenous GAPDH is used as a loading control. The mean expression ± SEM of CD81 and CD82 after shRNA silencing at days 3, 5, and 7 post-transductions are reported on the graphs below (*n* = 4 independent experiments). (**C**) Luciferase activity of HTLV-1-LTR Luc reporter T-cells co-cultured with WT C91-PL cells, shRNAs-transduced C91-PL cells, or WT Jurkat T-cells, as a measure of HTLV-1 cell-to-cell transmission efficiency. Results were obtained from four independent experiments performed in triplicate with different lentivector productions. The histogram shows the mean luciferase activity ± SEM for each condition in %, normalized to the positive control (JKT-LTR-Luc co-cultured with WT C91-PL cells). Ordinary one-way analysis of variance statistical tests were used to assess any significant difference: ns = *P*-value >0.05; * = *P*-value ≤0.05; ** = *P*-value ≤0.01; *** = *P*-value ≤0.001; **** = *P*-value ≤0.0001.

Biofilm polarization is hypothesized to be an important parameter for the bulk delivery of HTLV-1 particles through cell-to-cell contacts. To test this hypothesis, the efficiency of viral transmission was assessed by putting reporter Jurkat T-cells in co-culture with C91-PL cells silenced or not for the expression of CD81 or CD82 (Fig. 6A). Reporter Jurkat T-cells (JKT-LTR-Luc) encode luciferase under the control of HTLV-1 long terminal repeat (LTR) promoter which is transactivated by the viral protein Tax during a productive infection (24). Hence, luciferase activity is relative to the efficiency of viral transmission. Of note, CD9 was excluded from the analysis at this point, as its silencing did not impact viral biofilm organization or HTLV-1 Env expression at the cell surface (Fig. 5B and G). Luciferase activity was measured after 24 hours of co-culture between reporter cells and CD81- or CD82-silenced C91-PL cells at days 3, 5, and 7 post-transductions. The efficiency of CD81 and CD82 silencing was controlled by western blot and reached up to 70% as compared to their expression in WT cells (Fig. 6B).

For each time point, we observed no significant difference in the luciferase activity of reporter cells after co-culture with WT C91-PL cells or CD81-silenced C91-PL cells (Fig. 6C). In contrast, luciferase activity was severely reduced upon co-culture with CD82-silenced C91-PL cells (Fig. 6C). Importantly, the infectivity of C91-PL cells correlated with CD82 silencing efficiency, demonstrating the direct impact of CD82 expression on HTLV-1 cell-to-cell transmission. For instance, the 67% ( ±6%) decrease in CD82 expression at day 3 post-transduction resulted in a 61% ( ±4%) reduction of HTLV-1 transmission, whereas a less efficient CD82 knockdown at day 7 post-transduction (around 43% ± 5%) led to a 49% ( ± 6%) reduction in infectivity (Fig. 6C). To control that viral budding was not differentially impacted by the loss of CD81 or CD82 expression, we quantified HTLV-1 Gag release by western blot upon CD81 and CD82 silencing (Fig. S6A). A small decrease in the viral release was noticed in both conditions as compared to the control (around 20%, Fig. S6B). This relative decrease was mainly due to an increased proportion of Gag retained in the cell lysate (Fig. S6C), supporting the hypothesis that CD81 and CD82 silencing might partially inhibit virion accumulation at the cell surface and release, which is consistent with the observations made in Fig. 5E. Nevertheless, the significant reduction in luciferase activity measured strictly upon CD82 silencing resulted from impaired cell-to-cell infectivity, which indicates a crucial role for CD82 tetraspanin in HTLV-1 intercellular transmission.

### N-glycosylation is a key parameter for the polarization of HTLV-1 biofilms

**Fig 7:**
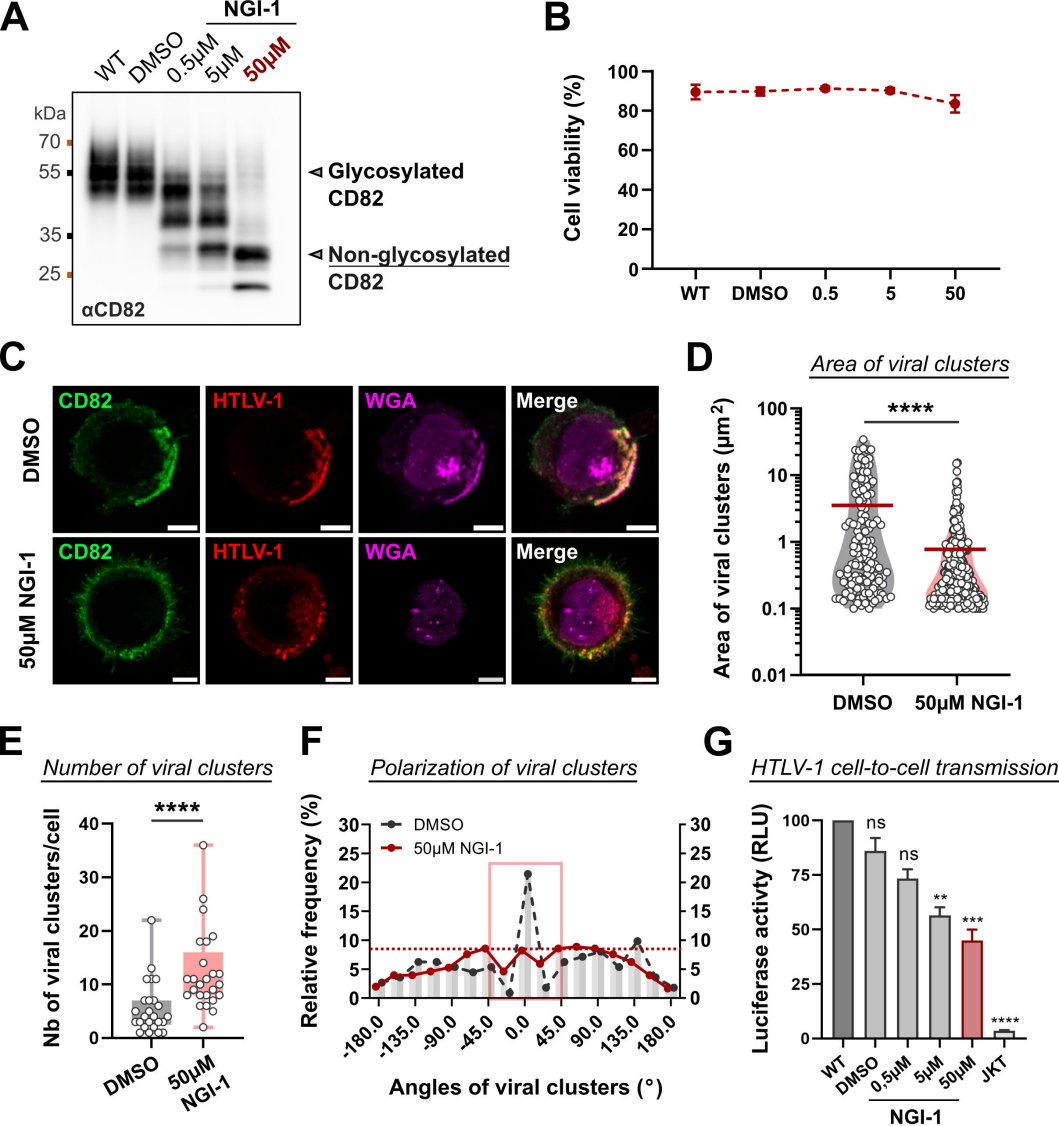
N-glycosylation is a key parameter for the polarization of HTLV-1 biofilms. (**A**) Representative immunoblot showing CD82 glycosylation profiles in C91-PL cells treated or not (WT/ dimethyl sulfoxide, DMSO) with different concentrations of NGI-1 (0.5 µM, 5 µM, or 50 µM) for 48 hours. (**B**) Viability of C91-PL cells ± SD (%) related to panel A. *n* = 3 independent experiments. (**C**) Representative confocal microscopy images of C91-PL cells treated or not with NGI-1 as in panel A. All cells were fixed and stained for CD82 (green), for HTLV-1 using serum from a HAMP-TSP patient (red), or for glycoproteins using WGA-conjugated lectins (magenta). Scale bars = 5 µm. (**D**) Violin plot showing the area (µm^2^) of individual viral clusters in C91-PL cells treated with DMSO or with 50 µM of NGI-1 (*n* > 140 clusters per condition). Mean = red bar. (**E**) Box plot showing the number of viral clusters at the surface of C91-PL cells treated with DMSO or with 50 µM of NGI-1 (*n* = 25 cells in each condition). (**F**) Frequency distribution of viral cluster angles at the surface of C91-PL cells treated or not with NGI-1 as in panel A (*n* = 25 cells; *n* > 140 HTLV-1 clusters per condition). While a peak between −45° and 45° (red square) is representative of a polarized profile, the absence of a peak indicates a depolarized pattern (red dotted line). (**G**) Luciferase activity of HTLV-1-LTR Luc reporter T-cells co-cultured with C91-PL cells treated or not (WT, DMSO) with different concentrations of NGI-1 (0.5 µM, 5 µM, or 50 µM), as a measure of HTLV-1 cell-to-cell transmission efficiency. Results were obtained from three independent experiments performed in triplicate. The histogram shows the mean luciferase activity ± SEM for each condition in %, normalized to the positive control (JKT-LTR-Luc co-cultured with WT C91-PL cells). Kruskal-Wallis statistical tests were used to assess any significant difference with the WT condition: ns = *P*-value >0.05; ** = *P*-value ≤0.01; *** = *P*-value ≤0.001; **** = *P*-value ≤0.0001.

To go further, we hypothesized that the differential effects observed for CD81 and CD82 on HTLV-1 biofilm polarization and cell-to-cell transmission could arise from their distinct three-dimensional structures. While CD81 and CD82 share conserved topologies, they differ in terms of post-translational modifications in their large extracellular loop (LEL): for example, CD82 LEL is highly N-glycosylated as opposed to CD81 LEL (48), especially in HTLV-1-infected T-cells (34). To test this hypothesis, we treated HTLV-1-infected T-cells (C91-PL) with different concentrations of an N-glycosylation inhibitor (NGI-1). Upon treatment, we obtained an effective deglycosylation of CD82, which was optimal for an NGI-1 concentration of 50 µM (Fig. 7A). Of note, we observed no significant impact on cell viability upon drug treatment (Fig. 7B). Then, C91-PL cells were treated with 50 µM of NGI-1 and surface-stained for CD82, HTLV-1, and surface glycoproteins using wheat germ agglutinin (WGA)-conjugated lectins (Fig. 7C). Upon NGI-1 treatment, the WGA signal was lost which confirms the effective deglycosylation of surface molecules (Fig. 7C). Also, NGI-1 treatment led to a complete reorganization of HTLV-1 biofilms (Fig. 7C): first, we noticed a significant decrease in the area of individual viral clusters, from a mean area of 3.7 µm^2^ in the control to a mean area of 0.8 µm^2^ per viral cluster in NGI-1-treated cells (Fig. 7D). This was combined with a significant increase in the number of viral clusters per cell, from a mean of six clusters per cell in the control to a mean of 12 clusters per cell upon NGI-1 treatment (Fig. 7E). Overall, the inhibition of N-glycosylation induced a complete depolarization of viral aggregates at the cell surface (Fig. 7F), similar to the phenotypes observed upon CD82 silencing (Fig. 5G). These results support the assumption that N-glycosylation of host cell membrane proteins, including CD82, is required for the polarization of HTLV-1 biofilms.

We then investigated the effects of N-glycosylation inhibition on HTLV-1 cell-to-cell transmission (Fig. 7G). To this aim, C91-PL cells were treated (or not) with different concentrations of NGI-1 (0 µM, 0.5 µM, 5 µM, or 50 µM) and co-cultured with JKT-LTR-Luc reporter cells (Fig. 7G). Our results show that global inhibition of N-glycosylation, including CD82, induces a 40%–50% reduction in the cell-to-cell transmission efficiency of HTLV-1 (Fig. 7G). This reduction appears to be dose-dependent and reaches its maximum at 50 µM of NGI-1. Altogether, our results suggest that N-glycosylation of surface molecules, including tetraspanin CD82, is required for the polarization of viral biofilms and their subsequent transfer through intercellular contacts.

## DISCUSSION

Polarized aggregates of infectious HTLV-1 virions, identified as biofilms, have been observed in HTLV-1-infected T-cell lines and at the surface of primary CD4(+) T-cells isolated from HAM/TSP patients or asymptomatic HTLV-1 carriers (24), suggesting a conserved role in HTLV-1 pathogenesis. Besides this pioneering discovery, the molecular and functional description of viral biofilms is still in its infancy, though of utmost importance to fully understand retroviruses pathogenesis. In this study, we explored the molecular composition of viral biofilms and identified specific cellular components (i.e.*,* tetraspanins) that control the cell-surface distribution and the intercellular transmission of HTLV-1 biofilms.

Here, we first demonstrated that CD9, CD81, and CD82 tetraspanins were enriched in HTLV-1 biofilms (Fig. 2) and clustered in their vicinity at the cell surface (Fig. 3). Interestingly, TEMs pre-exist in the plasma membrane of lymphocytes, where more than three different molecules of this family can reside in a single complex (49). It is noteworthy that several viruses budding from the plasma membrane like the herpes simplex virus 1 (50), the influenza A virus (IAV) (51), and HIV-1 (30, 52) assemble in TEMs. Therefore, TEMs containing CD9, CD81, and CD82 could be preferentially targeted by HTLV-1 structural proteins and act as exit platforms. Accordingly, earlier reports showed that both HTLV-1 Gag and Env proteins could interact with tetraspanins (33, 34). For instance, the HTLV-1 Gag matrix domain was reported to interact with the intracellular loops of both CD81 and CD82 when transiently over-expressed in HEK293T cells (33). Here, we showed that ectopic Gag-YFP expression in HTLV-1-infected T-cells deficient in virus production was sufficient to relocate CD81 and CD82 within Gag clusters at the cell plasma membrane (Fig. 3). This suggested that Gag is probably interacting with CD81 and CD82 in infected T-cells. However, our coimmunoprecipitation experiments in HTLV-1-infected T-cells only confirmed the interaction of Gag with CD82, suggesting that Gag might have more affinity for endogenous CD82 than for CD81 in infected T-cells (Fig. S5). Consequently, CD81 recruitment to Gag clusters might be bridged by CD82. In addition, CD82 could also be recruited by HTLV-1 Env, as it was reported that they both associate early along CD82 biosynthesis pathway trafficking up to the cell plasma membrane (34). This interaction was proposed to account for HTLV-1 Env localization and concentration in TEMs. Here, we propose that CD82 might form a complex with both Gag and Env (Fig. 5; Fig. S5), with Gag being a crucial determinant for the initiation of CD82 polarization (Fig. 3). Importantly, other viruses targeting TEMs recruit different tetraspanins. For instance, IAV specifically recruits CD81 to its assembly sites (51), and HIV-1 Gag and Env accumulate in TEMs (31, 52) mainly through interactions with CD81 (30). Interestingly, while HIV-1 Gag could also interact with CD63 and CD82 (30), HIV-1 Env did not interact with CD82 (34), highlighting different but specific molecular determinants involved in viral proteins-tetraspanins interplay.

Interactions between viral proteins and tetraspanins within TEMs frequently translate into their incorporation into budding virions. This was previously reported for several enveloped viruses such as the hepatitis C virus, IAV, and HIV-1 [for a review, see (53)]. For example, in addition to CD81 which interacts with HIV-1 Gag and Env (30, 52), HIV-1 particles incorporate CD9, Tspan14, CD53, CD63, and CD82 (54, 55). Similarly, we showed that CD9, CD81, and CD82 are incorporated into HTLV-1 particles (Fig. 4), suggesting that interactions between HTLV-1 structural proteins and tetraspanins are likely to be maintained through the budding process. Moreover, both CD81 and CD82 seem to play a role in HTLV-1 budding process as their silencing induces a reduction in Env expression at the cell surface (Fig. 5) along with a small decrease in viral release (Fig. S6). Likewise, HIV-1 release is decreased by 20% upon CD81 silencing which demonstrates that these tetraspanins promote retroviruses’ egress (31, 56). Taken together, these observations suggest that tetraspanin webs may facilitate Env glycoproteins uptake, HTLV-1 assembly, and budding during virus morphogenesis through the induction of large assembly platforms that concentrate both tetraspanins and viral proteins.

In addition to their potential function in HTLV-1 assembly, we showed that tetraspanins could modulate viral clustering and HTLV-1 biofilm polarization at the cell surface (Fig. 5). Examples of viruses that concentrate within TEMs in discrete plasma membrane regions were reported in the literature: for instance, IAV infection redistributes CD81 on the plasma membrane into concentrated patches of viral assembly and budding sites (51). Also, HIV-1 Gag expression induces the concentration of CD9 and CD81 nanoclusters within Gag assembly areas (57). Here, we observed that the transient expression of HTLV-1 Gag-YFP in infected T-cells that do not produce viral particles (nor biofilms) initiates CD81 and CD82 polarization within Gag clusters (with a similar but smaller effect for CD9). Taken together, this suggests that retroviral Gag proteins have the potential to drive tetraspanin clustering and polarization. Here, this phenomenon could be indirect for CD9/CD81 and direct for CD82, which can form complexes with HTLV-1 Gag (Fig. S5). Importantly, while CD9 or CD81 silencing had no effect on HTLV-1 biofilm organization at the cell surface, CD82 silencing induced a complete reorganization of viral aggregates (Fig. 5). Similar effects have been reported for HIV-1 where CD81 silencing induces a redistribution of HIV-1 Gag clusters at the surface of infected T-cells (30). Here, CD82, but not CD81, is required to maintain the architecture and polarization of HTLV-1 biofilm, highlighting distinct molecular events involved in HIV-1 and HTLV-1 clustering. This observation is consistent with the ability of Env and Gag viral proteins to interact preferentially with CD81 (for HIV-1) or CD82 for (HTLV-1). Consequently, the concomitant aggregation of CD82 and assembling Gag molecules into large platforms at the cell plasma membrane could promote HTLV-1 Gag-Env encounters leading to the production of dense biofilms containing mature particles. Comparable events were reported for HIV-1, where Gag assembly is known to induce the aggregation of small Env clusters into larger domains that are completely immobile (58).

The molecular mechanism ruling the CD82-dependent polarization of HTLV-1 biofilms remains to be elucidated. CD82 can form multimeric complexes with other tetraspanins but also with integrins and heparan sulfate proteoglycans (59–61), which are adhesion molecules also enriched in HTLV-1 biofilms (24). Thus, tetraspanins, which modulate intercellular adhesion processes (especially CD82 [62]) could direct virions assembly to biofilms preferentially formed at intercellular junctions (as shown in Video S1, Fig. S1). Additionally, the differential effects observed for CD9, CD81, and CD82 could arise from their three-dimensional structure and subsequent dynamics in the cell plasma membrane. These three tetraspanins, which share conserved topologies, differ in terms of post-translational modifications in their LEL. For example, CD82 LEL is highly N-glycosylated as opposed to CD9 and CD81 LELs (48), especially in HTLV-1-infected T-cells (34) where the MGAT-3 glycosyltransferase (also named beta-1,4-mannosyl-glycoprotein 4-beta-N-acetylglucosaminyltransferase) responsible for CD82 N-glycosylation (60) is up-regulated (GSE17718 database [42]). Interestingly, CD82 N-glycosylation is known to promote CD82-integrins interactions (60), modulate CD82 membrane clustering (63), and participate in tetraspanins complex formation (64). Moreover, increased glycosylation of extracellular domains is directly linked to a decrease in the diffusivity of transmembrane proteins (65). Accordingly, CD9 and CD81 dynamics are inferior to CD82 dynamics in the plasma membrane of non-tumoral human breast epithelial (HB2) cells (66). Therefore, we can speculate that CD82 N-glycosylation could promote its interaction with membrane or submembrane components (i.e., integrins) and restrict its dynamics at the cell surface. Both processes could thus lead to a subsequent decrease in the local diffusion of partner molecules, which could ultimately contribute to the pivotal role of CD82 in the maintenance of HTLV-1 biofilm architecture. Interestingly, after treating HTLV-1-infected T-cells with an inhibitor targeting the oligosaccharyltransferase, which is responsible for initiating N-glycosylation of membrane proteins, we observed CD82 deglycosylation along with a complete depolarization of viral biofilms and a significant reduction in viral transmission efficiency (Fig. 7). Therefore, we propose that N-glycosylation of host cell membrane proteins, including CD82, is required for HTLV-1 biofilm polarization and infectivity. This is in line with the work of Mazurov and collaborators who showed that the expression of highly O-glycosylated CD43 and CD45 molecules on the surface of T-cells favored the assembly of viral particles into large structures and promoted HTLV-1 intercellular transmission (45).

Finally, we demonstrated that the CD82-dependent clustering of viral particles within TEMs at the cell surface is a key parameter in HTLV-1 cell-to-cell transmission (Fig. 6). Early electron microscopy studies revealed that other viruses could also form viral aggregates at the cell surface resembling viral biofilms, including IAV (67), vesicular stomatitis virus (68), and HIV-1 (56, 69). Clusters of mature HIV-1 particles resembling biofilms were also detected at the surface of infected MOLT cells (human acute lymphoblastic leukemia, T-cells) (30), at the surface of primary CD4(+) T-cells upon *in vitro* HIV-1 infection (69, 70), and at the surface of CD4(+) T-cells from HIV-1-infected patients (69). Virions aggregation significantly increases the chances of multiple viral genomes being jointly delivered to target cells, as shown recently for the vesicular stomatitis virus (VSV) (71) and HIV-1 (69), which demonstrates the ability of enveloped viruses to establish collective infectious units. Accordingly, viral biofilms account for 80% of HTLV-intercellular transmission *in vitro* (24), and our results further support the importance of the collective transmission of clustered virions. Moreover, our results provide a molecular basis for this process, as CD82 silencing, which provokes HTLV-1 biofilm reorganization, severely reduces the ability of infected T-cells to transmit the virus (by up to 60%, Fig. 6). In contrast, CD81 silencing, which does not affect HTLV-1 biofilm clustering (Fig. 5), has no significant effect on HTLV-1 cell-to-cell transmission (Fig. 6). Altogether, our results suggest that viral biofilm polarization is required for efficient viral cell-to-cell transmission. Different mechanisms which are not fully deciphered yet may account for the initiation and maintenance of virus polarization in different viral infections. For HTLV-1, our overall observations provide a specific role for CD82 in the regulation of HTLV-1 biofilm polarization and its subsequent intercellular transmission.

## MATERIALS AND METHODS

### Cell lines

C91-PL cells (HTLV-1 chronically infected T-cell line, Cellosaurus, ref CVCL_0197), MT-2 cells (HTLV-1 chronically infected T-cell line [72], National Institutes of Health [NIH], ref 237), and C8166 cells (HTLV-1-infected T-cells deficient in virus production [44], ECACC ref 88051601) were obtained from Helene Dutartre’s lab and cultured in 75 cm^2^ flasks in biosafety level 3 (BSL-3) facilities at 37°C with 5% CO_2_. Of note, C8166-infected T-cells express the Tax accessory protein but encode a Rex-deficient mutant: nuclear export of non-spliced viral RNA is thus impaired. Consequently, this cell line does not express HTLV-1 Env or Gag structural proteins and has no viral genomic RNA available for packaging. As such, these cells are not producing any viral particles and are BF(−). Jurkat cells (non-infected T-lymphocytes, from ATCC, ref ACC 282) were also cultured in 75 cm^2^ flasks in BSL2/BSL3 facilities at 37°C with 5% CO_2_. Jurkat cells transfected with a plasmid encoding the luciferase enzyme (Luc) under the control of HTLV-1 LTR promoter, which is transactivated by HTLV-1 Tax (Jurkat-LTR-Luc [24]), were grown under hygromycin selection (450 µg/mL, Invivogen). All T-lymphocyte cell lines were maintained in complete RPMI medium: RPMI GlutaMAX (ThermoFisher Scientific) supplemented with decomplemented fetal calf serum (FCS) 10% (ThermoFisher Scientific) and 1% penicillin/streptomycin (Merck). Human embryonic kidney 293T cells were maintained in DMEM GlutaMAX (Dulbecco’s modified Eagle medium, ThermoFisher Scientific) supplemented with 10% decomplemented FCS (ThermoFisher Scientific) and 1% penicillin/streptomycin (Merck) at 37°C with 5% CO_2_.

### Plasmids and DNA constructs

The plasmid encoding HTLV-1 Gag-YFP under the cytomegalovirus (CMV) promoter (pHTLV Gag-YFP) was described previously (73) and is a kind gift from Dr. David Derse’s lab. HTLV-1 Gag-YFP is expressed as a fusion protein: it was reported that its intracellular trafficking is similar to WT Gag and that its cellular expression efficiently allows HTLV-1 virus-like particle production (35). The pFG12 GFP(+) vectors encoding the control shRNAs and shRNAs targeting CD81 were a kind gift from Dr. Birke Bartosch’s group (Inserm U1052, Cancer Research Centre of Lyon, France). Plasmids encoding shRNAs targeting CD9 and CD82 were designed in the lab as follows: shCD9 and shCD82 synthetic sequences (designed and ordered on GeneArt, ThermoFisher Scientific) were placed downstream the U6 promoter and cloned between XbaI and HpaI restriction sites into the pFG12 GFP(+) lentiviral vector (obtained from Dr. David Baltimore’s lab, Addgene plasmid #14884). All constructs were verified by DNA sequencing (Eurofins genomics, Plasmidsaurus). The packaging vectors pVSV-G (encoding the vesicular stomatitis virus envelope glycoprotein) and p8.2 (encoding HIV-1 Gag-Pol and its accessory proteins except for Vpu) were used for vectorization (as it was previously described [74, 75]) of the different shRNAs into lentiviral particles (see below).

### Antibodies

All antibodies used in this study along with their targets, species, clone numbers, references, initial concentrations, and working dilutions are listed in Table S1.

### Electroporation of HTLV-1 chronically infected cells for microscopy

Electroporation was conducted using the Gene Pulser Xcell Electroporation System (BioRad), according to the following protocol: cells were washed in 1× phosphate buffered saline (PBS, ThermoFisher Scientific), counted, and resuspended in Opti-MEM (1×) Reduced Serum Medium (ThermoFisher Scientific) at a concentration of 2 × 10^7^ cells per milliliter. Cells (8 × 10^6^; i.e.*,* 400 µL) were then mixed with 10 µg plasmid for each condition. The resulting mixtures were put at 37°C for 30 min and transferred to 0.4 cm electroporation cuvettes (Gene Pulser/MicroPulser, BioRad), which were submitted to electroporation using 10 ms pulses at 180V repeated three times at 1-second intervals. Cells were then gently transferred to six-well plates in pre-warmed RPMI GlutaMAX (ThermoFisher Scientific) 10% FCS without penicillin/streptomycin (P/S) and cultured at 37°C with 5% CO_2_. After 24–48 hours, cells were washed in 1× PBS and plated on 0.01% poly-L-lysine-coated (Sigma-Aldrich) glass-bottom Fluorodishes (World Precision Instruments[WPI]) for 30 min at room temperature. Samples were then fixed using 4% paraformaldehyde for 15 min at room temperature.

### Living cells imaging

Living C91-PL cells electroporated with HTLV-1 Gag-YFP construct were plated in uncoated six-well plates in complete RPMI medium 20 hours post-electroporation. Plates were placed in the incubator chamber of an automated laser-scanning confocal microscope (Cell-Discoverer 7 LSM900, Zeiss, Germany). Epifluorescence images were generated every 15 min for 5 hours (Video S1; Fig. S1).

### Isolation of viral biofilms for mass spectrometry or microscopy

Viral biofilms were isolated from the supernatants of chronically infected cell lines (wild-type for mass spectrometry or electroporated with HTLV-1 Gag-YFP for microscopy). Cells were cultured in 25 cm^2^ flasks in RPMI complete medium at an initial concentration of 0.5 × 10^6^ cells per milliliter. Cultures were left untouched for 96 hours to favor biofilm enrichment and its release in the culture supernatant. Cells were removed from the culture by centrifugation at 1,000 *× g* for 5 min. Supernatants were collected and then submitted to a 10,000 *× g* centrifugation for 40 min. Pellets containing biofilms were resuspended and used immediately. For microscopy experiments, pellets were resuspended in 1× TNE (10 mM Tris/HCl pH 7.4, 100 mM NaCl, 1 mM EDTA), plated on 0.01% poly-L-lysine-coated (Sigma-Aldrich) glass-bottom Fluorodishes (WPI) for 30 min at room temperature, and fixed using 4% paraformaldehyde for 15 min. For mass spectrometry experiments, 10,000 *g* pellets containing biofilms were resuspended in 1× PBS and inactivated at 56°C for 30 min.

### Atomic force microscopy coupled with fluorescence imaging

Biofilms isolated from Gag-YFP(+) expressing C91-PL cells were diluted in 1× TNE (Tris/HCl pH 7.4, NaCl, EDTA) and plated on glass-bottom Fluorodishes (WPI) coated with 0.01% poly-L-lysine (Sigma-Aldrich). AFM imaging was performed on a NanoWizard IV atomic force microscope (Bruker) at room temperature. Fluorescence imaging was performed by wide-field illumination using a Nikon Ti-U inverted microscope mounted on the AFM machine and equipped with a 100×, 1.4 NA oil objective (Nikon). The AFM tip position was calibrated with the optical image using a specific software module (DirectOverlay, JPK Bio-AFM, Bruker). AFM topographic images were acquired in quantitative imaging mode using BL-AC40TS cantilevers (Olympus). Cantilevers’ sensitivity and spring constant (kcant  =  0.1 N/m) were calibrated using the thermal noise method. The force applied to the samples was kept at 200 pN with an approach/retract speed of 25 µm/s (z-range = 100 nm). For image analysis, we used the JPK-data processing software (Bruker). Images were flattened with a histogram line fit and minor noise was removed using low-pass Gaussian and median filtering. Z-color scales (Fig. 1D) are shown as relative after processing. To measure the maximal central height of each particle, we used the cross-section tool of the analysis software.

### Mass spectrometry experiments

Biofilms isolated from C91-PL cells were diluted in buffer A (0.1% formic acid). Samples were injected for in-line analysis using nano-flow high-performance liquid chromatography (RSLC U3000, ThermoFisher Scientific) coupled to a mass spectrometer equipped with a nano-electrospray source (Q Exactive HF, ThermoFisher Scientific). Peptides were separated on a capillary column (0.075 mm × 500 mm, Acclaim Pepmap 100, reverse phase C18, NanoViper, ThermoFisher Scientific) according to a 2%−40% buffer B gradient (0.1% formic acid, 80% acetonitrile) at a flow rate of 300 nL/min during 128 min. Spectra were recorded using the Xcalibur 4.1 software (ThermoFisher Scientific) with the 128LowQtt.meth method. Spectral data of the three independent experiments were analyzed using MaxQuant v2030 and Perseus v161043, with the use of the leading FPP v3.4 script. As a reference database, we used: RefProteome_HUMAN-cano_2022_01_UP000005640.fasta and UniProt-taxonomy 11908_202111.fasta (human T-cell leukemia virus type 1) with the following fixed modification: carbamidomethylation (C) and the following variable modifications: acetyl (protein N-term); oxidation (M). Proteins identified with at least two peptides were kept. Also, proteins matching with a “Contaminants” database (keratin, trypsin, etc.) were removed from the analysis. The whole experimental process was conducted by Serge Urbach and Mathilde Decourcelle (Montpellier FPP platform).

### Mass spectrometry results analysis

Raw mass spectrometry data were filtered to remove “contaminants” including mitochondrial and ribosomal proteins, and the 100 most abundant proteins were selected based on their IBAQ score (which is the intensity averaged by the number of peptides that can be detected for a given protein). To only keep candidates detected with high confidence, molecules identified with less than three peptides were excluded from the analysis. The remaining 69 candidates were then matched against a list of proteins known to be up-regulated by HTLV-1 infection (GSE17718 database [42]) and compared to potent HTLV-1 biofilm components identified in the literature.

### shRNA-carrying lentivectors production and transduction

HEK293T cells (3 × 10^6^) were plated in 10 cm culture dishes and cultivated overnight. Cells were then co-transfected using CaCl_2_/2× HEPES buffered saline (HBS) with plasmids (see “Plasmids and DNA constructs”) encoding VSV-G envelope (pVSV-G), HIV-1 Gag-Pol (p8.2), scrambled shRNAs, or shRNAs directed against the following targets: CD9, CD81, or CD82 (shRNAs sequences are listed in Table S1). Culture media containing lentiviral particles were collected 48 hours post-transfection and filtered through 0.45 µm membranes. Particles were then purified by ultracentrifugation at 100,000 *× g* for 1 hour and 30 min at 4°C on a 25% sucrose cushion. Pellets were resuspended in 1× TNE (Tris/HCl pH7.4 NaCl, EDTA). The concentration of particles was determined by Videodrop (Myriade). C91-PL cells were transduced with the shRNA-carrying lentivectors using a total of 100 particles per cell. Twenty-four-hour post-transduction cells were washed and cultured in RPMI complete medium for 72 hours (D3), 120 hours (D5), and 168 hours (D7). Then, cultures were centrifuged at 1,000 *g* for 5 min. Cells were collected, washed, and resuspended in 1× PBS at a concentration of 1 million cells per milliliter. Five hundred microliters of cells was plated on 0.01% poly-L-lysine-coated (Sigma-Aldrich) glass-bottom Fluorodishes (WPI) for immunofluorescence or resuspended in RIPA lysis buffer for western blots (see below). Supernatants were collected and centrifuged at 10,000 *× g* for 40 min to pellet biofilms. Biofilms-containing pellets were resuspended in 1× PBS and plated on glass-bottom Fluorodishes (WPI) for immunofluorescence or resuspended in PBS 0.2% Triton for western blots (see below).

### Immunofluorescence for confocal microscopy

Fixed C91-PL cells (WT, transduced with shRNAs, or electroporated with Gag-YFP) plated on fluorodishes were incubated in 50 mM NH_4_Cl for 5 min at room temperature (RT) to quench free aldehydes and in 1× PBS containing 3% bovine serum albumin (BSA, Sigma-Aldrich) for 15 min at RT to block non-specific epitopes. After washing with PBS 3% BSA, samples were stained using 1 µg/mL of primary mouse antibodies (αEnvgp46, αCD9, αCD81, or αCD82; see Table S1 for references) or rabbit antibodies (αCD43) diluted in PBS 3% BSA for 1 hour at RT. After several washes with PBS 3% BSA, samples were incubated with 1 µg/mL of secondary antibodies (AlexaFluor647 anti-mouse, ThermoFisher, or Rabbit-Atto647N anti-rabbit, Sigma-Aldrich) for 1 hour at RT. Finally, samples were washed with 1× PBS and stored at 4°C in 2 mL PBS until acquisition. Confocal images were obtained using a laser-scanning confocal microscope (LSM980, Zeiss) equipped with a 63*×*, 1.4 NA oil objective. All images were processed using ImageJ software (Fiji).

### Confocal image analysis

For the calculation of PCC in Fig. 1B, we used z-projections of whole cells (fixed regions of interest[ROIs]) that were analyzed using the JaCoP plugin of the ImageJ software (Fiji). This coefficient ranging from −1 to +1 describes the linear relationship between the gray levels of the two channels: if the fluorescence intensity in one channel always gets brighter when the other does, then Pearson’s coefficient = 1. If one channel gets dimmer when the other gets brighter, then Pearson’s coefficient = −1. For colocalization analyses in Fig. 3F, a schematic representation detailing the segmentation process and the calculation of the area of intersection between the two channels is provided in Fig. S3C. To analyze Env clusters at the surface of HTLV-1-infected cells transduced with scrambled shRNAs or shCD9/shCD81/shCD82 in Fig. 5, we used ImageJ software (Fiji). Background noise was excluded using a mask in which only intensities above [Min + 0.2 * (Max − Min)] were kept (Min: minimum pixel intensity, Max: maximum pixel intensity). After thresholding, the area of each cluster was extracted using the “Analyze particles” command on ImageJ (Fig. 5C). The number of viral clusters per image (and thus per cell) after thresholding was used to quantify the number of Env clusters per cell (Fig. 5D). The (total) MFI of the Env signal per image (and thus per cell) was extracted using ImageJ without thresholding (Fig. 5E). For polarization analyses, an automated macro (considering the cell as a circle) attributed an angle between −180° and 180 to each Env cluster encountered on the cell surface with respect to the largest one positioned at 0° (representative images in Fig. 5F). The frequency distribution of all angles (Fig. 5G) was then obtained using Prism software (GraphPad).

### Immunofluorescence for 2D STED super-resolution microscopy

Fixed isolated biofilms and/or whole C91-PL cells (electroporated with Gag-YFP) plated on 0.01% poly-L-lysine-coated (Sigma-Aldrich) Fluorodishes were incubated in 50 mM NH_4_Cl for 5 min at RT to quench free aldehydes; incubated in PBS 3% BSA supplemented with 0.05% saponin for 15 min at RT to block non-specific epitopes and permeabilize the cells; and washed once using the same buffer. Then, cells were stained for 1 hour and 30 min at RT with primary mouse antibodies (αEnvgp46, αCD9, αCD81, or αCD82), rabbit antibodies (αYFP), or actin-staining dyes (STAR red phalloidin, Abberior) diluted in PBS 3% BSA with 0.05% saponin. After three washes with PBS with 0.05% saponin and 3% BSA, samples were incubated with the appropriate secondary antibodies (STAR Orange anti-rabbit, STAR Orange anti-mouse, or STAR Red anti-mouse, Abberior) diluted in PBS 3% BSA with 0.05% saponin for 1 hour and 30 min at RT in the dark. Finally, samples were washed three times with 1× PBS and stored at 4°C in 2 mL PBS until acquisition. Dual-color STED 2D images were acquired on a STED super-resolution microscope (Abberior Instruments Expert Line GmbH) equipped with a 100× oil objective using 580 nm (Star-Orange) and 630 nm (Star-Red) excitation laser sources, coupled with a pulsed 775 nm STED laser. Using 25% of STED laser power, we could obtain a lateral resolution of less than 100 nm. All images were processed with ImageJ software (Fiji).

### Transmission electron microscopy

HTLV-1 chronically infected T-cells (C91-PL) were fixed using 1% glutaraldehyde, 4% paraformaldehyde (Sigma-Aldrich), in 0.1 M phosphate buffer (pH 7.2) for 24 hours. Then, samples were washed in PBS and post-fixed using 2% osmium tetroxide (Agar Scientific) for 1 hour. Cells were then fully dehydrated in a graded series of ethanol solutions and propylene oxide. They were impregnated with a mixture of (1:1) propylene oxide/Epon resin (Sigma-Aldrich) and left overnight in pure resin. Samples were then embedded in Epon resin (Sigma-Aldrich), which was allowed to polymerize for 48 hours at 60°C. Ultra-thin sections (90 nm) of these blocks were obtained with a Leica EM UC7 ultramicrotome (Wetzlar, Germany). Sections were stained with 2% uranyl acetate (Agar Scientific) and 5% lead citrate (Sigma-Aldrich), and observed under a transmission electron microscope (JEOL 1011).

### Immuno-electron microscopy assay

HTLV-1 chronically infected T-cells (C91-PL) were fixed using 4% paraformaldehyde in 1× PBS for 2 hours, washed twice with 1× PBS, and centrifuged for 10 min at 300 *× g*. Cell pellets were embedded in 12% gelatin and infiltrated overnight with 2.3 M sucrose at 4°C. Ultra-thin cryosections (90 nm) were obtained using a Leica FC7 cryo-ultramicrotome at −110°C. Sections were retrieved in a mix of 2% methylcellulose supplemented with 2.3 M sucrose (1:1) and deposited onto formvar-/carbon-coated nickel grids. Sections were incubated at 37°C to remove gelatin and labeled with primary antibodies targeting HTLV-1 Env, CD9, CD81, or CD82. Then, grids were washed with 1× PBS and labeled with secondary antibodies coupled to 6 nm-diameter gold nanoparticles. Grids were washed using 1× PBS, post-fixed with 1% glutaraldehyde, and rinsed in distilled water. Contrast staining was achieved by incubating the grids with a mix of 2% uranyl acetate supplemented with 2% methylcellulose (1:10). Treated sections were then imaged under a transmission electron microscope (JEOL 1011).

### Western blot analysis

Before immunoblotting, HTLV-1 chronically infected cells (C91-PL) were washed in 1× PBS, pelleted at 1,000 *× g* for 5 min, and lysed in RIPA buffer (Sigma-Aldrich). The total protein concentration in cell lysates was measured using a Bradford protein assay kit (ThermoFisher). Biofilm fractions isolated from cell culture supernatants were resuspended in PBS 0.2% Triton (Sigma-Aldrich). Proteins from cell lysates (20 µg per condition) or biofilm fractions (identical volume in each condition) were loaded on 10% acrylamide gels and separated by SDS-PAGE. Separated proteins were transferred onto polyvinylidene difluoride membranes (ThermoFisher Scientific) using wet transfer with tris-glycine-methanol buffer (milli-Q H_2_O supplemented with 15% methanol and 10% 10× Tris-glycine solution, Euromedex). Then, membranes were incubated for 30 min in Tris-buffered saline-Tween (TBS-Tween) solution (milli-Q H_2_O 10% Trizma base-HCl 1M pH8 supplemented with 0.1% Tween 20, Sigma-Aldrich) supplemented with 5% milk to saturate non-specific sites and incubated with the corresponding primary antibodies (αGagp19, αCD9, αCD81, or αCD82) overnight at 4°C. Blotting membranes with antibodies targeting endogenous GAPDH was used as a loading control for cell lysates. After several washes in 5% milk TBS-Tween, membranes were incubated for 2 hours at room temperature with horse radish peroxidase (HRP)-conjugated αMouse antibodies (Dako). After washing with TBS-Tween, HRP activity was revealed using ECL Prime reagents (Amersham). Images were acquired on a Chemidoc Imaging system (BioRad). Each band intensity was measured using the ImageJ software. Quantification of viral release was established by comparing the intensity of the Gagp19 signal detected in biofilms to the Gagp19 signal in both fractions (CL: cell lysates or BF: biofilms) with the following formula: [Gagp19 BF/(Gagp19 BF + Gagp19 CL)] * 100. Each time point was normalized to its corresponding control condition. Quantification of tetraspanin release was determined using the same process, except that each condition was corrected by its dilution factor (DF): BF * DF/(BF * DF + CL * DF).

### Detection of molecular complexes by immunoprecipitation

C91-PL and C8166 cells were harvested as explained in the Western blot analysis section. Pellets were lysed using the following buffer: 50 mM Tris-HCl pH = 7.4, 150 mM NaCl, 1 mM EDTA, 1 mM CaCl_2_, 1 mM MgCl_2_, 1% CHAPSO, protease inhibitors (lysis buffer). Five hundred microgram of total proteins was incubated overnight with or without 1 µg of anti-tetraspanins antibodies (αCD9, αCD81, αCD82; see Table S1) on a rotator at 4°C. The remaining lysates were kept and stored at −20°C (input). Then, antibody-protein complexes were deposited on 25 µL of protein A magnetic beads (Dynabeads protein A, Life Technologies) and incubated for 4 hours at 4°C on the rotator. Beads were washed five times using 200 µL of lysis buffer. Then, the buffer was removed and 20 µL of 4× Laemmli buffer was added to the beads. For western blot analysis, samples were heated at 95°C for 10 min and loaded onto a 10% SDS-PAGE gel.

### NGI-1 drug treatment

C91-PL cells (500,000 cells) were seeded in 12-well plates and incubated with or without different concentrations of NGI-1 for 48 hours at 37°C. Concentrations of NGI-1 tested: 0 µM (DMSO only), 0.5 µM, 5 µM, or 50 µM. Following drug treatment, cell viability was measured using trypan blue exclusion counting. Cells were then collected, washed, and resuspended in 1 mL of 1× PBS: 500 µL of cells were fixed and plated on 0.01% poly-L-lysine-coated (Sigma-Aldrich) glass-bottom Fluorodishes (WPI) for immunofluorescence analysis, and the remaining 500 µL was resuspended in 60 µL of RIPA lysis buffer for western blotting (see above).

### Viral transmission to T-cells

For cell-to-cell transmission assays upon tetraspanin silencing: Jurkat reporter T-cells (JKT-LTR-Luc, 50,000 cells) were plated in 96-well plates and co-cultured with HTLV-1-infected T-cells (10,000 cells) transduced or not (WT) with tetraspanins-specific shRNAs, or with non-transduced control cells (Jurkat, 10,000 cells) for 24 hours. For cell-to-cell transmission assays upon N-glycosylation inhibition (NGI-1 treatment): Jurkat reporter T-cells (JKT-LTR-Luc, 50,000 cells) were plated in 96-well plates and co-cultured with HTLV-1-infected T-cells (10,000 cells) treated or not (WT, DMSO) with different concentrations of NGI-1 (0.5 µM, 5 µM, or 50 µM), or with untreated control cells (Jurkat, 10,000 cells) for 24 hours. Cell viability was measured using trypan blue exclusion counting. Reporter cells’ luciferase activity was determined using the luciferase reporter assay system (Promega) following the manufacturer’s instructions. Luciferase activity was measured using a 96-microplate luminometer (Mithras or GloMax), and the background signal due to leaky luciferase activity of Jurkat-LTR-Luc cells was subtracted. Results were presented as normalized to the control (JKT-LTR-Luc co-cultured with WT C91-PL cells) in %.

### Statistical analyses

Statistical tests were performed with Prism software (GraphPad), using Kruskal-Wallis or ordinary one-way analysis of variance tests for multiple comparisons. Significant differences were represented as follows: ns = *P*-value > 0.05; * = *P*-value ≤ 0.05; ** = *P*-value ≤ 0.01; *** = *P*-value ≤ 0.001, and **** = *P*-value ≤ 0.0001.

## SUPPLEMENTAL MATERIAL

Figure S1 mbio.01326-23-s0001.tif Time-lapse of Gag-YFP+ biofilms formation in living chronically infected T cells.

Figure S2 mbio.01326-23-s0002.tif STRING interaction network of cellular components identified in isolated HTLV-1 biofilms by mass spectrometry.

Figure S3 mbio.01326-23-s0003.tif HTLV-1 Gag is sufficient to initiate the polarization of tetraspanins at the surface of T cells.

Figure S4 mbio.01326-23-s0004.tif Organization of HTLV-1 biofilms at the surface of infected T cells and incorporation of CD9, CD81, and CD82 into viral particles.

Figure S5 mbio.01326-23-s0005.tif CD82 forms a complex with HTLV-1 Gag.

Figure S6 mbio.01326-23-s0006.tif Impact of CD81 and CD82 shRNA silencing on HTLV-1 biofilm release.

Video Legend mbio.01326-23-s0007.docx Legend for Video S1.

Table S1 mbio.01326-23-s0008.tif Antibodies and shRNAs used in this study.

Table S2 mbio.01326-23-s0009.tif Cellular proteins identified in HTLV-1 biofilms by mass spectrometry.

Video S1 mbio.01326-23-s0010.avi Polarization of Gag-YFP+ biofilms in living chronically infected T cells.

ASM does not own the copyrights to Supplemental Material that may be linked to, or accessed through, an article. The authors have granted ASM a non-exclusive, world-wide license to publish the Supplemental Material files. Please contact the corresponding author directly for reuse.
