# Supplementary figures and images for: HTLV-1 biofilm polarization maintained by tetraspanin CD82 is required for efficient viral transmission
Source: mBio. 2023 Oct 27;14(6):e01326-23. doi: 10.1128/mbio.01326-23 (PMC10746275; doi:10.1128/mbio.01326-23)

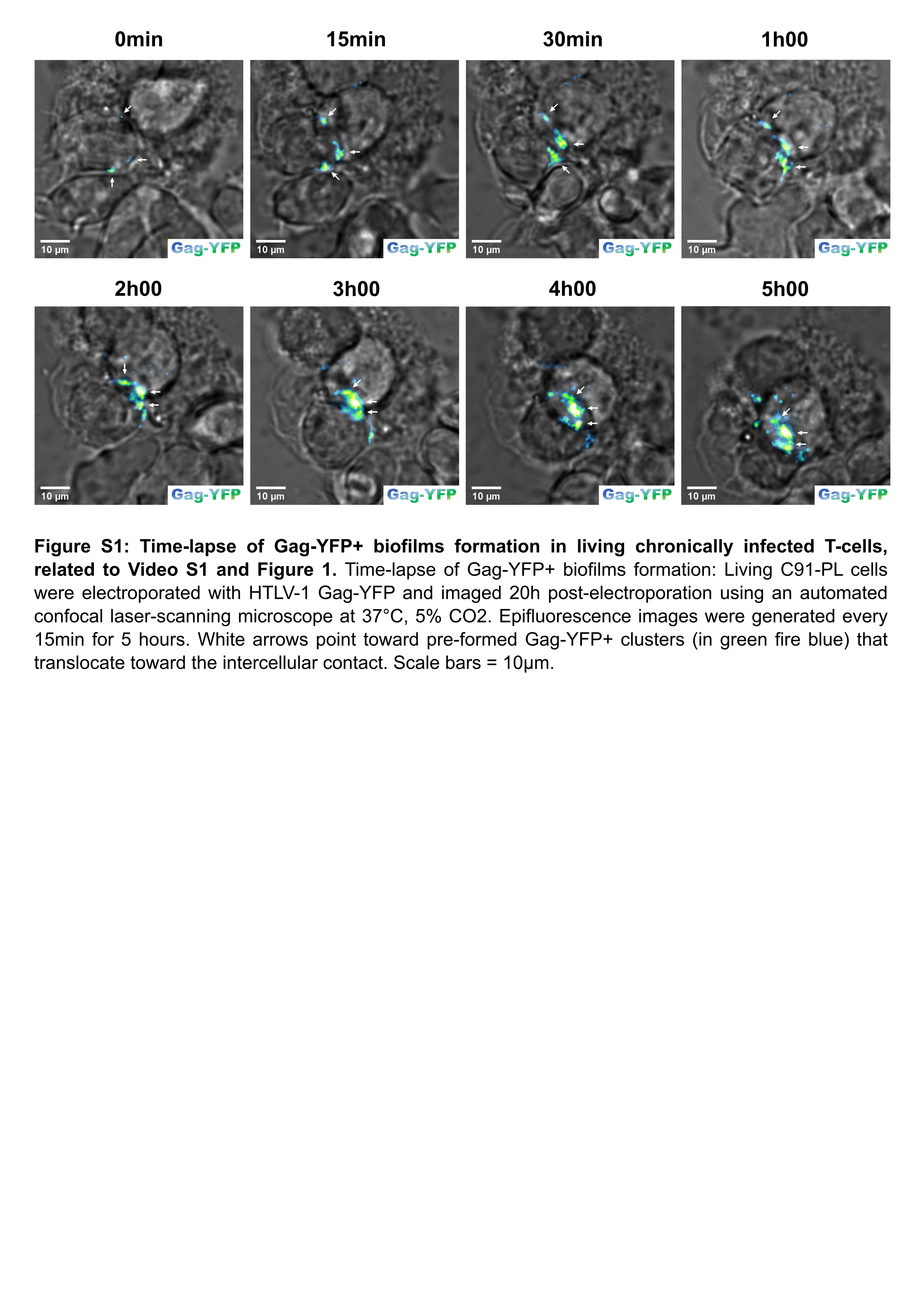

Supplement: Figure S1 — Time-lapse of Gag-YFP+ biofilms formation in living chronically infected T cells. [file mbio.01326-23-s0001.tif]

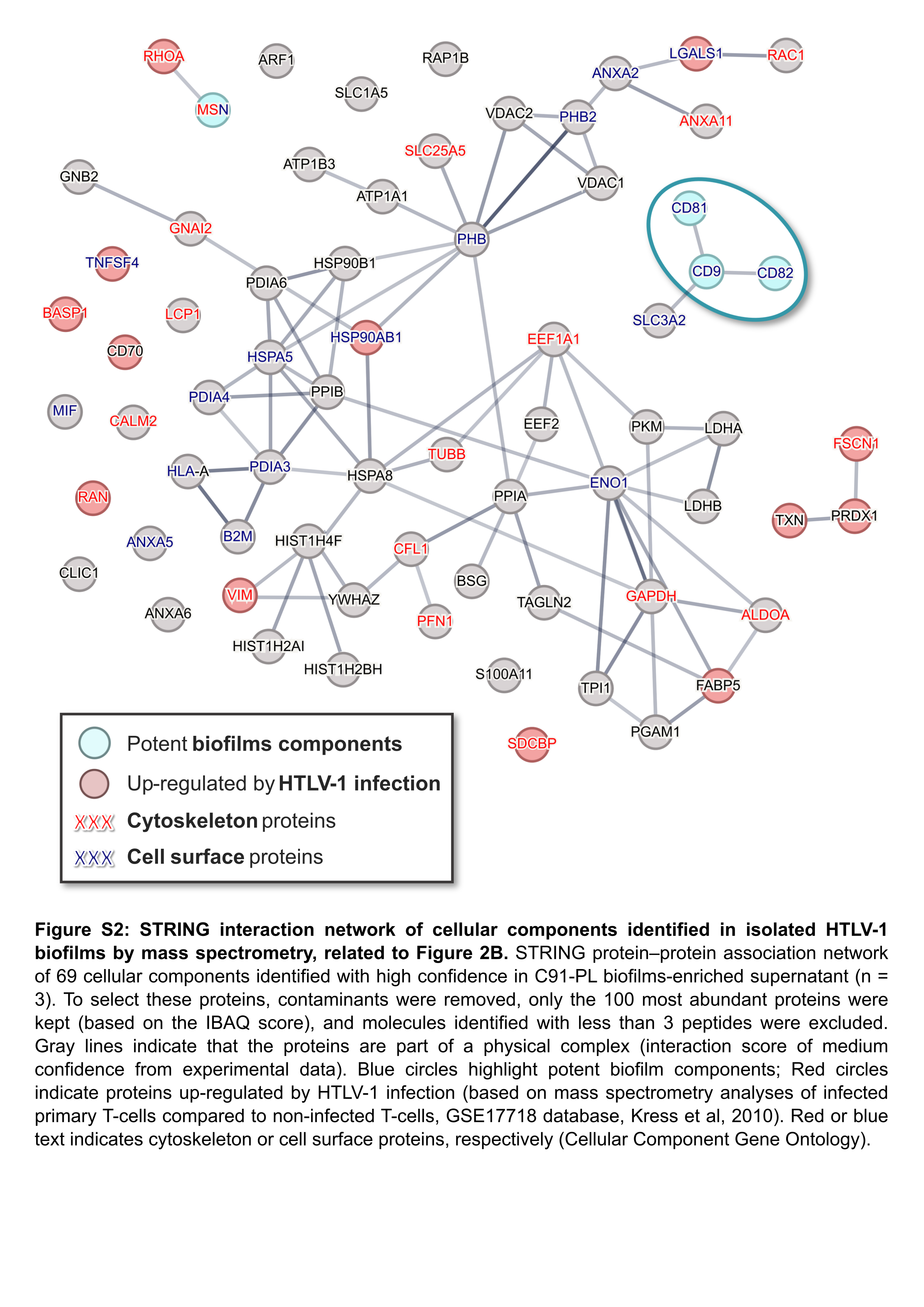

Supplement: Figure S2 — STRING interaction network of cellular components identified in isolated HTLV-1 biofilms by mass spectrometry. [file mbio.01326-23-s0002.tif]

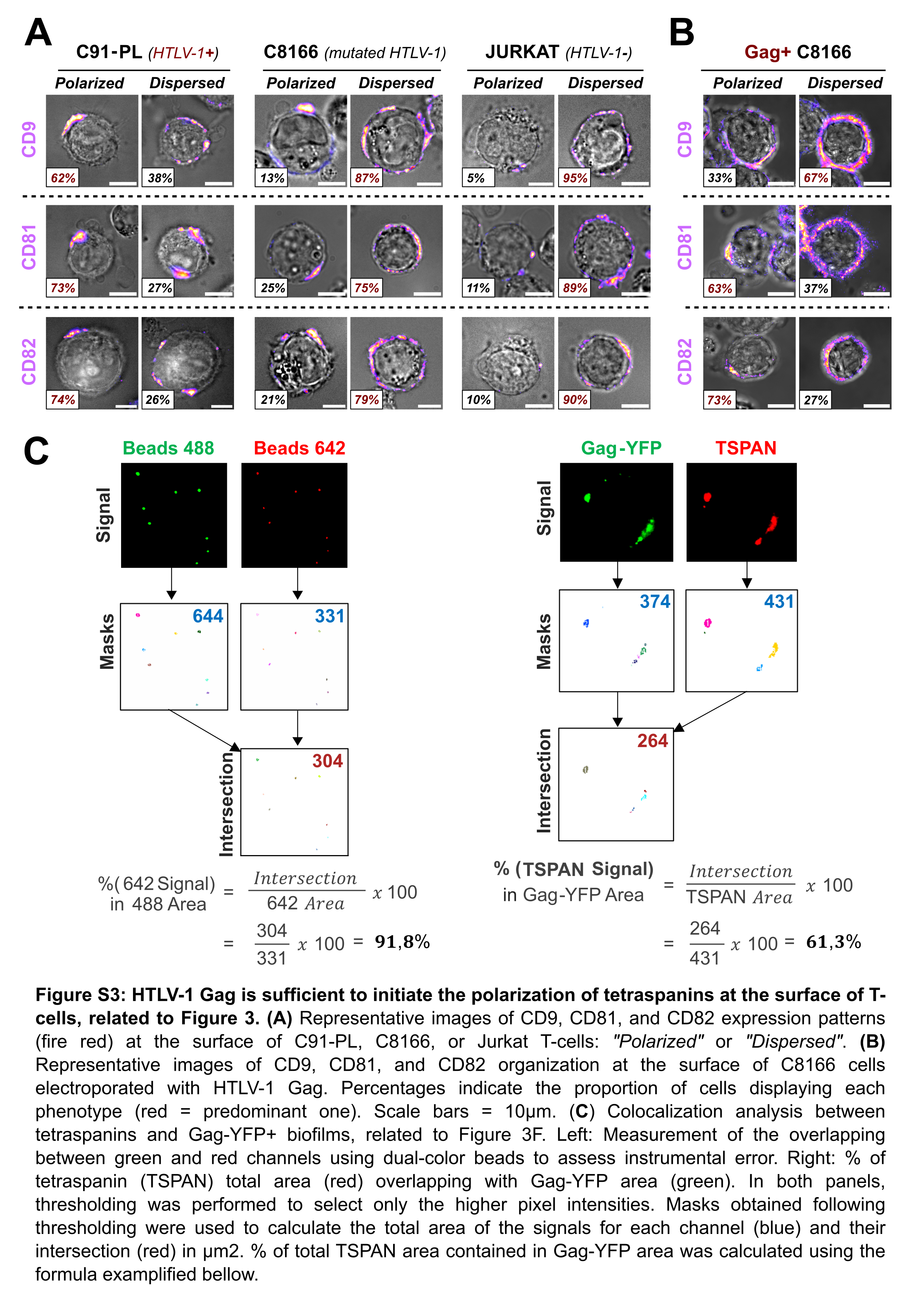

Supplement: Figure S3 — HTLV-1 Gag is sufficient to initiate the polarization of tetraspanins at the surface of T cells. [file mbio.01326-23-s0003.tif]

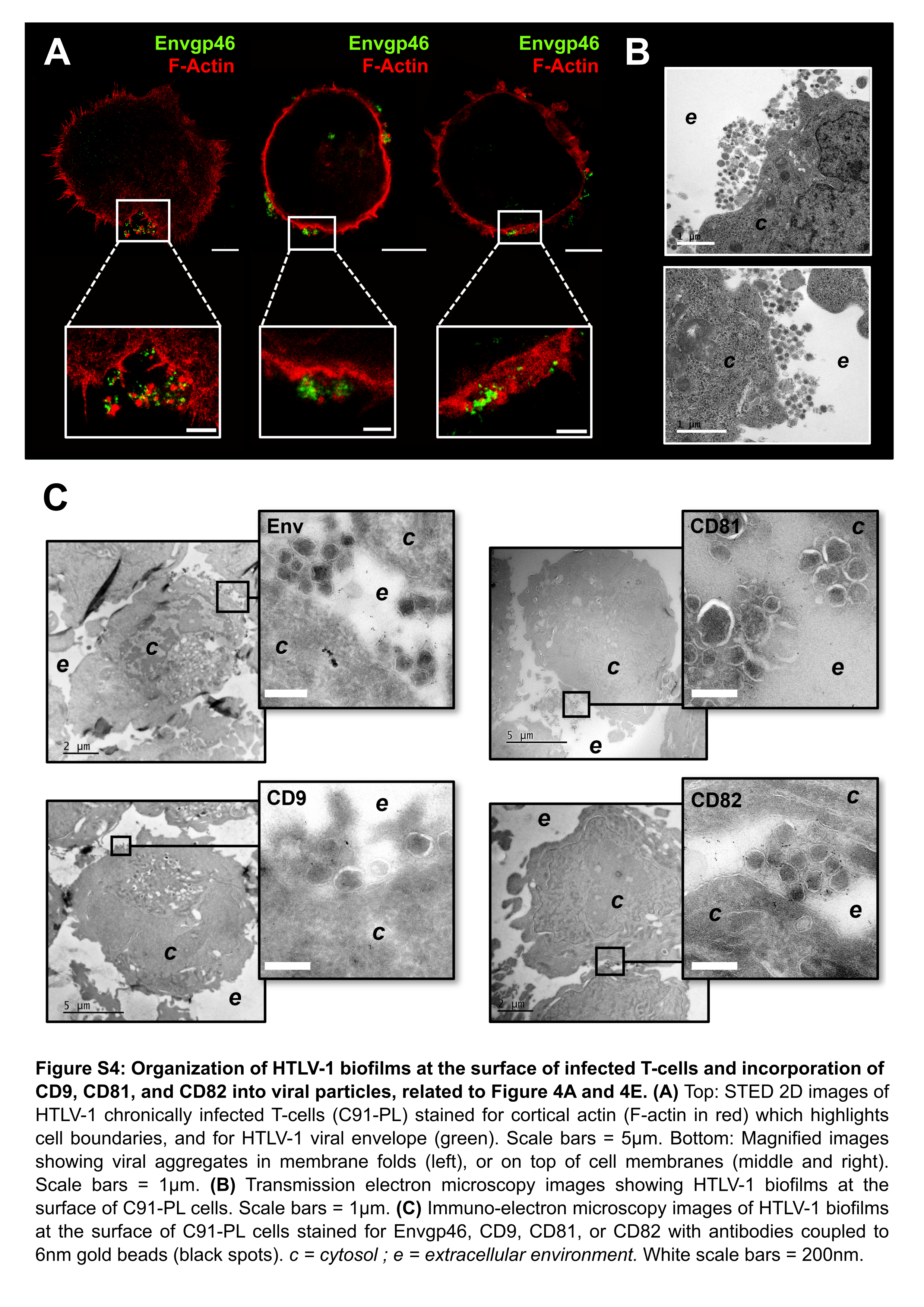

Supplement: Figure S4 — Organization of HTLV-1 biofilms at the surface of infected T cells and incorporation of CD9, CD81, and CD82 into viral particles. [file mbio.01326-23-s0004.tif]

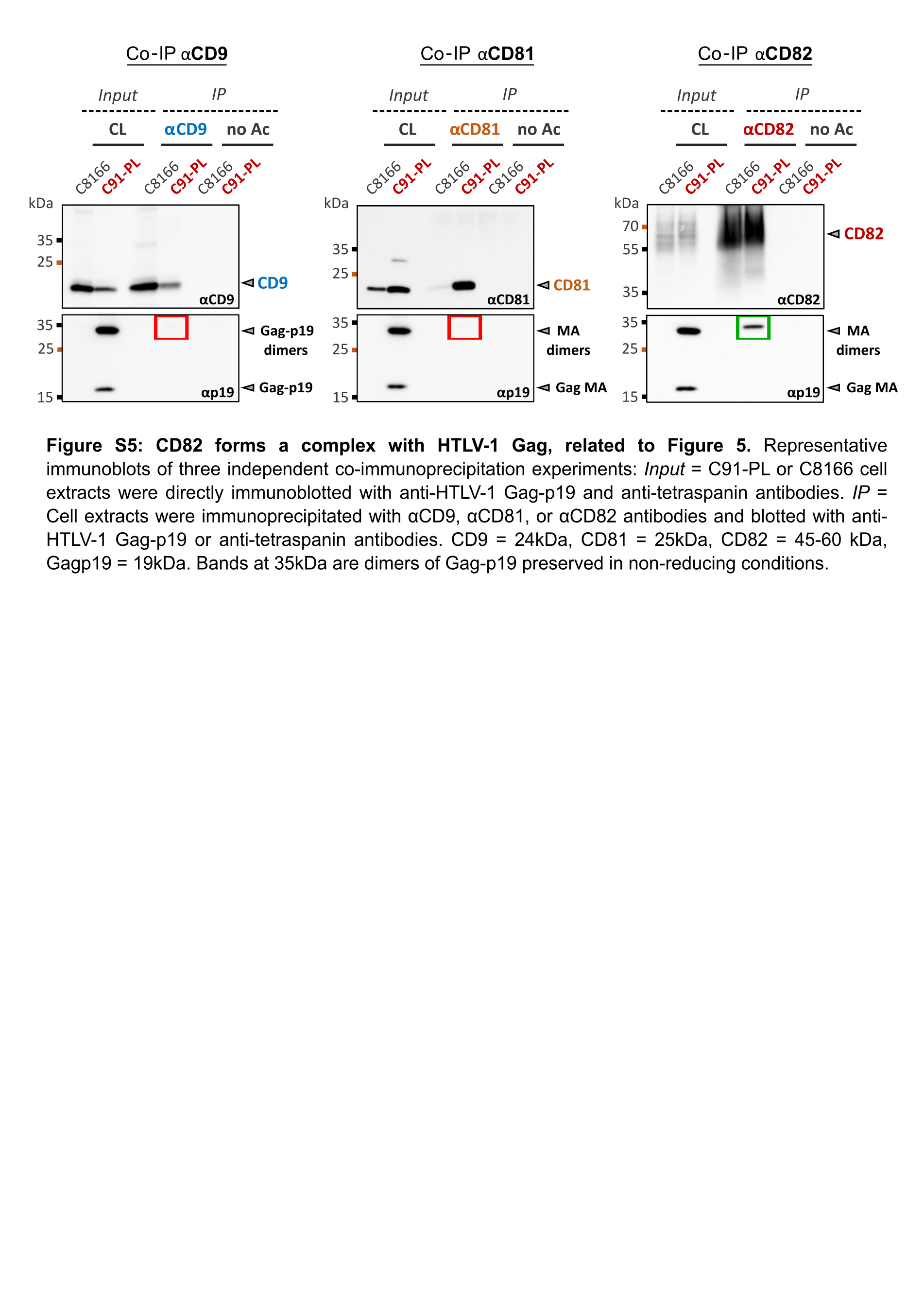

Supplement: Figure S5 — CD82 forms a complex with HTLV-1 Gag. [file mbio.01326-23-s0005.tif]

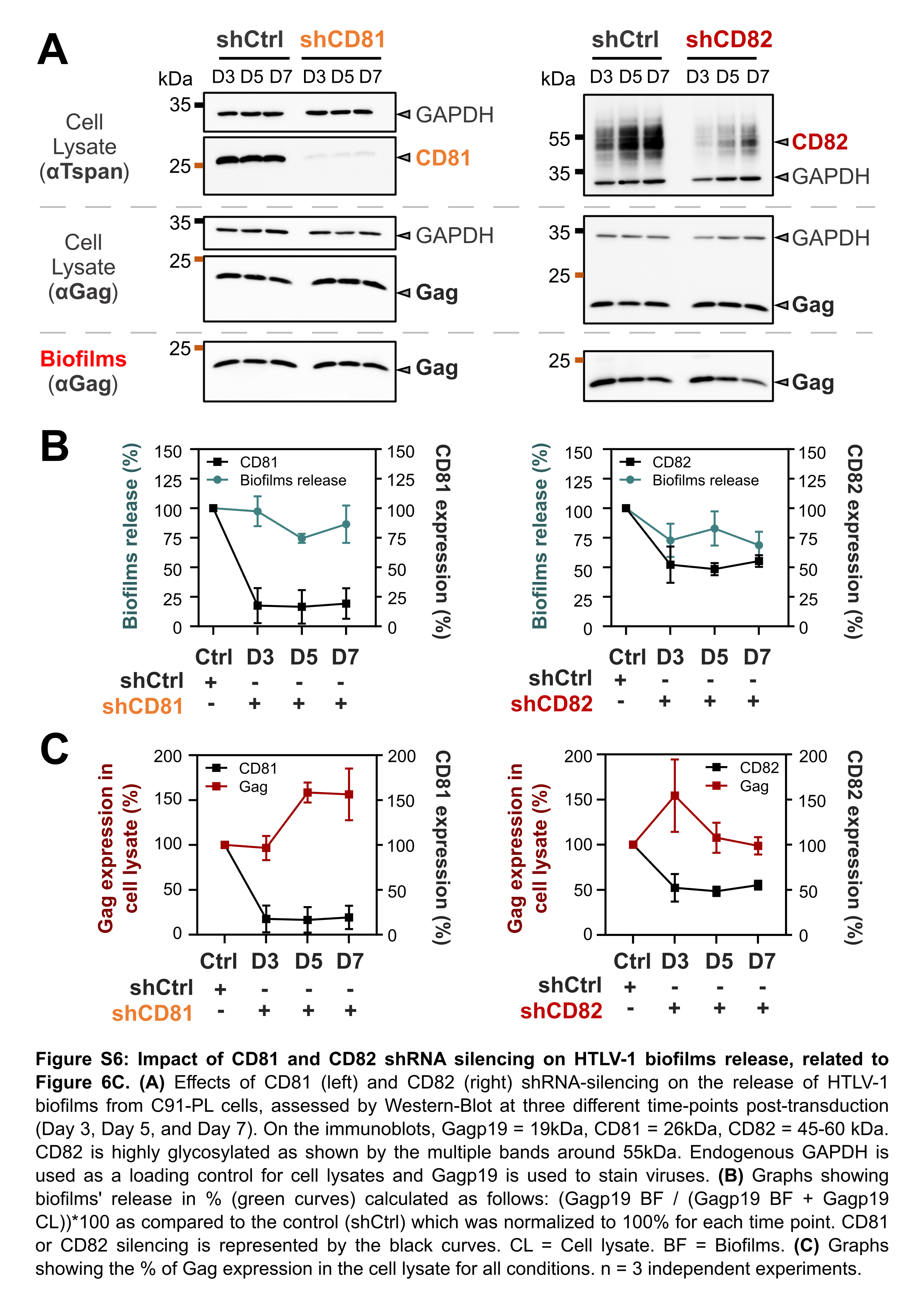

Supplement: Figure S6 — Impact of CD81 and CD82 shRNA silencing on HTLV-1 biofilm release. [file mbio.01326-23-s0006.tif]

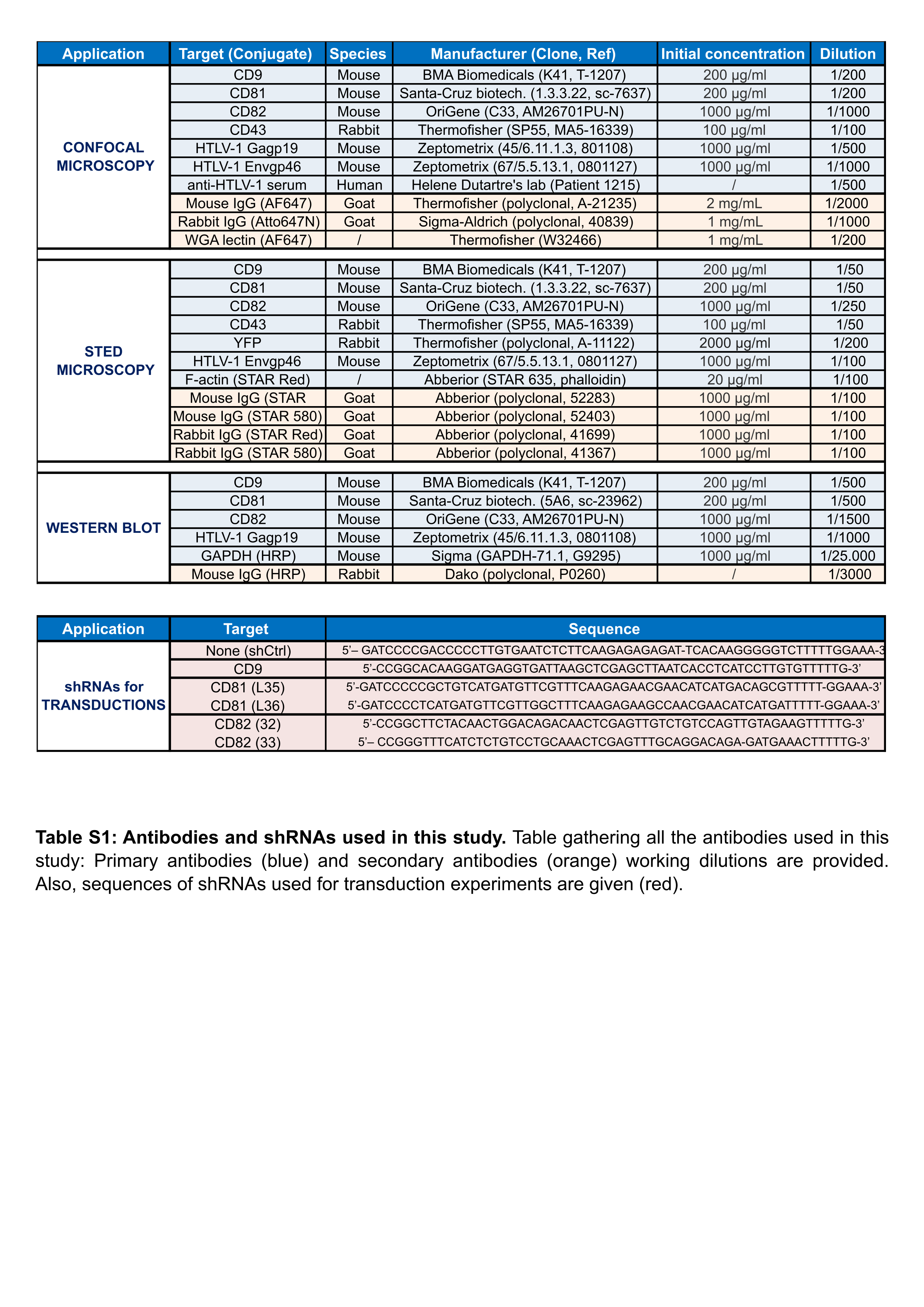

Supplement: Table S1 — Antibodies and shRNAs used in this study. [file mbio.01326-23-s0008.tif]

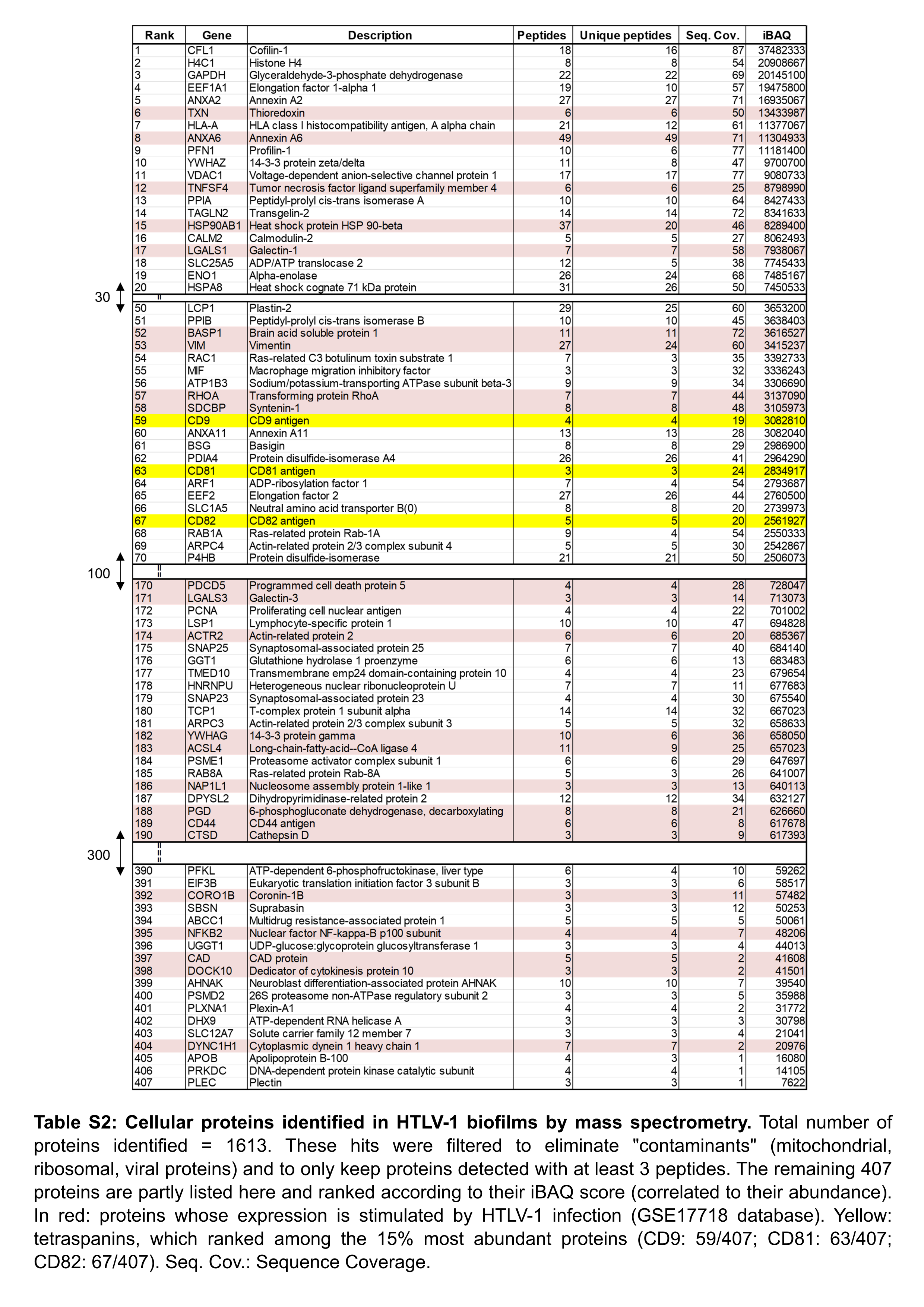

Supplement: Table S2 — Cellular proteins identified in HTLV-1 biofilms by mass spectrometry. [file mbio.01326-23-s0009.tif]
