# Supplementary material for: HTLV-1 biofilm polarization maintained by tetraspanin CD82 is required for efficient viral transmission
Source: mBio. 2023 Oct 27;14(6):e01326-23. doi: 10.1128/mbio.01326-23 (PMC10746275; doi:10.1128/mbio.01326-23)
Supplement: Video Legend — Legend for Video S1. [file mbio.01326-23-s0007.docx]

Supplemental Video S1

**Video S1: Polarization of Gag-YFP+ biofilms in living chronically infected T-cells, related to Figure 1.** Time-lapse microscopy imaging of living C91-PL cells electroporated with HTLV-1 Gag-YFP, 20 hours post-electroporation. Sequential epifluorescence images were generated every 15min for 5 hours. Arrows indicate pre-formed Gag-YFP(+) clusters that polarize toward the cell-to-cell junction. Scale bar = 10µm.
